# Mixed-anion engineering of ASnS_2_O (A = Ba, Sr, Ca): first-principles insights into structure–property tuning and water-redox band-edge screening

**DOI:** 10.1039/d6ra07784f

**Published:** 2026-11-13

**Authors:** Golam Morshed, S. M. Anowarul Haque, Abdul Wahed, Joyonta Das, Md Sabbir Hossain, Mohammad Sefat, Fahmid Hasan Fahim, Tawsif Md Samiul Hossain

**Affiliations:** a Department of EEE, Mymensingh Engineering College (University of Dhaka) Mymensingh Bangladesh riyamahmed6350@gmail.com

## Abstract

Mixed-anion engineering provides a route to tune coupled structural and functional properties in Sn-based oxysulfides. Using density functional theory (GGA-PBE), we investigate ASnS_2_O (A = Ba, Sr, Ca) with a common ordered S/O substitution motif and selected alternative configurations. Structural relaxation yields triclinic P1 structures, with unit-cell volumes decreasing by 11.6% from 446.001 Å^3^ (Ba) to 394.341 Å^3^ (Ca). The primary configurations exhibit indirect band gaps of 1.461, 1.727, and 1.928 eV, respectively, while alternative anion orderings produce substantial variations. Fixed-volume calculations and Mulliken overlap populations indicate that volume contraction alone cannot explain band-gap widening. The valence-band edges are predominantly S/O-p-derived, whereas the conduction-band edges are mainly Sn-derived. Polycrystalline-averaged optical spectra reveal absorption maxima of 3.04 × 10^5^, 2.35 × 10^5^, and 2.56 × 10^5^ cm^−1^ at 18.8, 23.6, and 27.8 eV, respectively, beyond the visible region. Positive-definite elastic stiffness matrices support elastic mechanical stability, although mechanical responses are non-monotonic and strain-sensitive; CaSnS_2_O exhibits the highest calculated hardness (6.148 GPa). Sn-5p PBE+*U* calculations preserve the band-gap ordering without establishing absolute-gap accuracy. At pH = 0, nominal PBE–Mulliken estimates indicate a qualitative improvement in water-redox band-edge alignment from Ba to Ca, with BaSnS_2_O closest to the oxidation threshold. However, the configurations lie above selected competing decomposition assemblages, and *Γ*-point phonon calculations cannot establish full dynamical stability. Further assessment of phase stability, absolute band edges, carrier transport, and surface kinetics is required before practical photocatalytic feasibility can be established.

## Abstract 2

Using a common S/O motif, DFT shows cell contraction and gap widening from Ba to Ca in ASnS_2_O. DOS reveals S/O–p valence and Sn-derived conduction edges; optical and elastic responses vary, while water-redox alignment is screened qualitatively.

## Graphical abstract

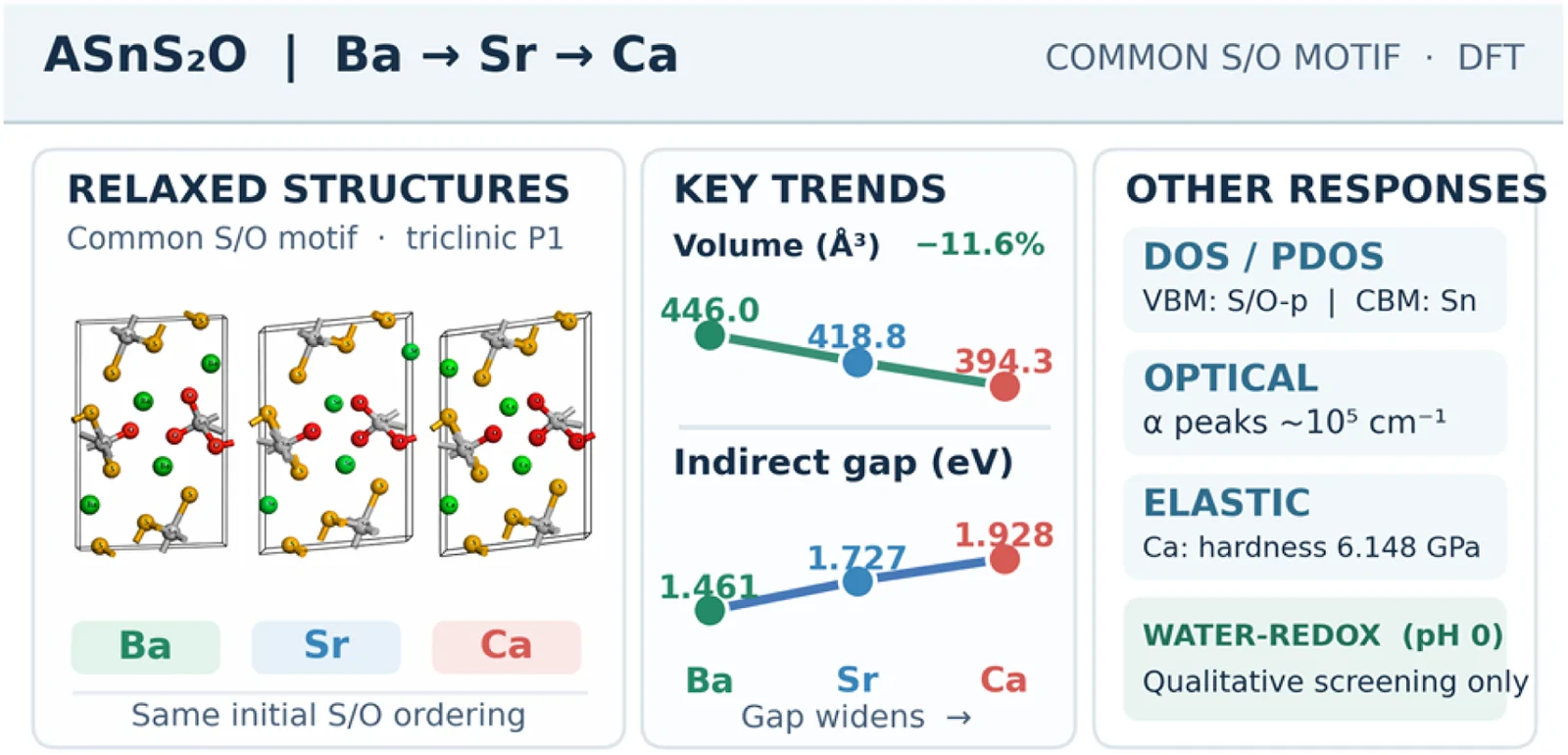

## 1. Introduction

The development of environmentally benign, chemically robust, and compositionally tunable semiconductors remains central to next-generation energy-conversion and optoelectronic technologies. Although metal-halide perovskites exhibit outstanding optoelectronic performance, concerns regarding Pb toxicity and long-term stability continue to motivate alternative inorganic semiconductors. Chalcogenide perovskites have consequently emerged as an important materials family because several members combine earth-abundant chemistry, strong optical absorption, tunable band gaps, and comparatively robust all-inorganic frameworks. Their potential for photovoltaic and related optoelectronic applications has been established through theoretical and experimental studies.^1–6^

Chalcogenide perovskites are commonly described by the ABX_3_ framework, where cation chemistry, crystallographic distortion, and the chalcogen sublattice can substantially modify the electronic structure. Previous studies have demonstrated band-gap control through structural and chemical tuning and highlighted the sensitivity of optical and carrier-related properties to composition.^7–10^ Progress in phase-stability analysis, thin-film growth, colloidal synthesis, and lower-temperature processing further emphasizes the connection between composition, structure, and realizability.^11–16^

Within this family, Sn-based chalcogenides are particularly attractive because replacing transition-metal B-site species such as Zr or Hf with post-transition-metal Sn changes the orbital character near the band edges. First-principles studies of ASnS_3_ and ASnSe_3_ (A = Ba, Sr, and Ca) show that their frontier states are governed mainly by the Sn–chalcogen framework and that their electronic and optical properties are sensitive to composition and structural configuration.^17–20^ Experimental synthesis of CaSnS_3_ thin films further demonstrates the physical accessibility of Sn-containing sulfides,^21^ while related studies of sulfide and selenide perovskites reveal composition-dependent electronic, optical, mechanical, and transport behavior.^22,23^

Chemical substitution and alloying therefore offer effective routes for tuning chalcogenide perovskites without abandoning their parent structural chemistry. Alloying in BaZrS_3_ has been shown to modify lattice characteristics and band gaps, demonstrating the coupling between chemical modification and electronic structure.^8,24^ Anion chemistry provides an additional degree of freedom. Comparative S/Se studies of Sn-based chalcogenide perovskites demonstrate anion-dependent electronic energetics and carrier dynamics.^17^ These observations provide a physical basis for extending anion engineering beyond conventional sulfide–selenide substitution toward mixed O/S compositions, where the stronger chemical contrast between the two anions may produce substantial local structural and electronic rearrangements.

O/S mixing is especially interesting because O and S differ in ionic size, electronegativity, orbital energy, and bonding preference. According to Shannon's effective ionic radii, *r* (O^2−^, CN = 6) = 1.40 Å and *r* (S^2−^, CN = 6) = 1.84 Å, indicating that S^2−^ is approximately 31% larger than O.^2–25^ This substantial size mismatch provides a quantitative structural basis for local relaxation and may contribute to anion-ordering tendencies in mixed O/S environments. Their simultaneous presence can therefore alter local coordination, lattice distortion, orbital hybridization, and band-edge energetics. Vonrüti and Aschauer^26^ demonstrated that anion ordering significantly influences the structural energetics and electronic properties of perovskite oxysulfides. In particular, they reported that trans anion ordering is energetically favored over cis ordering by 0.23 and 0.33 eV per formula unit in BaZrOS_2_ and BaZrO_2_S, respectively, and identified pronounced ordering-associated polar distortions. Broader computational studies have also identified sulfur-rich oxysulfide perovskites as a promising design space for solar-energy conversion and photocatalytic screening.^27^ This concept is particularly relevant to ASnS_3_ because states near its fundamental gap are strongly associated with the Sn–S framework; partial replacement of S by O may therefore reorganize the local Sn–anion environment and frontier electronic states.

The importance of anion configuration has been further demonstrated through systematic first-principles investigations. Pilania *et al.*^28^ examined 246 symmetry-distinct anion configurations of CaHf(O_0.5_S_0.5_)_3_ within a 20-atom supercell and reported configuration-dependent band gaps ranging from 1.52 to 2.75 eV. These findings demonstrate that substantial electronic variations can arise from different anion arrangements even at fixed chemical composition. Structural-framework selection is another important consideration. Carr *et al.*^29^ investigated the polymorphic stability of 81 ABS_3_ compositions and reported that the needle-like NH_4_CdCl_3_-type structure is energetically preferred for approximately 77% of the investigated compounds. These studies emphasize the importance of both anion ordering and structural polymorphism in evaluating chalcogenide materials. In the present study, the same ordered S/O substitution motif was employed across the Ba-, Sr-, and Ca-containing ASnS_2_O models to enable an internally consistent comparison of A-site-dependent properties. The Ca-containing model was constructed from the SrSnS_3_ framework through Sr-to-Ca substitution rather than from the structurally distinct CaSnS_3_ phase discussed by Basera and Bhattacharya.^18^ Nevertheless, DFT evaluation of only the selected configurations does not establish the global configurational ground state, while an exhaustive comparison of competing structural polymorphs remains beyond the scope of this study.

Mixed-anion oxysulfide chemistry is also relevant to photocatalytic and photoelectrochemical water splitting. In addition to suitable photon absorption, a semiconductor must provide band-edge energetics compatible with proton reduction and water oxidation, while practical activity further depends on charge separation, recombination, surface kinetics, cocatalysts, and chemical stability.^30,31^ Visible-light-driven water reduction and oxidation have been demonstrated for Ti-based oxysulfides such as Sm_2_Ti_2_S_2_O_5_ and related Ln_2_Ti_2_S_2_O_5_ compounds,^32,33^ and overall water splitting has subsequently been achieved using Y_2_Ti_2_O_5_S_2_ under optimized reaction conditions.^34^ Subsequent studies highlighted the importance of charge trapping and recombination dynamics in Y_2_Ti_2_O_5_S_2_ (ref. 35) while its particulate formation mechanism has also been investigated in detail.^36^ These results do not establish equivalent photocatalytic activity for ASnS_2_O, but they strongly motivate examination of its intrinsic band-edge alignment as an initial thermodynamic screening criterion.

Despite substantial progress on parent chalcogenide perovskites, Sn-based ASnS_3_ compounds, and mixed-anion oxysulfides, the coupled consequences of ordered S/O incorporation and Ba → Sr → Ca A-site variation remain insufficiently established for ASnS_2_O. Variation of the A-site cation changes ionic size and lattice dimensions, introducing a chemical-pressure-like structural perturbation,^25^ whereas O incorporation simultaneously alters local Sn–anion coordination and bonding.^26^ Their combined action may therefore affect crystallographic distortion, orbital overlap, band-edge energetics, optical response, elastic behavior, and redox characteristics. A comparative Ba–Sr–Ca investigation within the same nominal ASnS_2_O stoichiometry thus provides a useful framework for identifying composition-dependent structure–property trends and examining how A-site variation interacts with mixed-anion coordination.

Accordingly, the present work investigates mixed-anion ASnS_3−*x*_O_*x*_ (*x* = 1), hereafter denoted as ASnS_2_O (A = Ba, Sr, and Ca), using first-principles density functional theory. To enable an internally controlled Ba–Sr–Ca comparison, a common structural-framework strategy was adopted, and the same ordered S→O substitution motif was applied across the A-site series, followed by full structural relaxation. To further assess the influence of anion ordering, additional S/O configurations were systematically enumerated and screened, followed by DFT evaluation of selected candidates. The optimized systems are examined through lattice geometry, local Sn–S/O coordination, simulated X-ray diffraction, electronic band structures, orbital-resolved densities of states, and polycrystalline-averaged optical spectra supplemented by frequency-dependent dielectric tensor components. The complete set of 21 independent triclinic elastic constants and derived mechanical parameters is evaluated to assess elastic stability and rigidity, while Mulliken electronegativity-based band-edge estimates are compared with the H^+^/H_2_ and O_2_/H_2_O redox levels to examine the qualitative water-redox band-edge alignment. This integrated analysis aims to clarify how mixed-anion coordination and A-site variation jointly regulate the structural, electronic, optical, mechanical, and redox characteristics of ASnS_2_O and to establish a theoretical basis for further photocatalytic and photoelectrochemical assessment.

## 2. Computational details

All first-principles calculations were performed within the framework of density functional theory (DFT) using the Cambridge Serial Total Energy Package (CASTEP) implemented in BIOVIA Materials Studio 2020.^37^ The BaSnS_3_ and SrSnS_3_ parent structures were obtained from the Materials Project database (mp-1183370 and mp-1205419, respectively).^38^ BaSnS_2_O was constructed from BaSnS_3_, whereas both SrSnS_2_O and CaSnS_2_O were derived from the SrSnS_3_ parent structure. For CaSnS_2_O, the Sr atoms were replaced by Ca. An alternative CaSnS_2_O configuration derived from the *Pnma* CaSnS_3_ framework was also optimized and lies 18.46 meV per formula unit above this SrSnS_3_-derived configuration (Table S12, SI). The mixed-anion ASnS_3−*x*_O_*x*_ (A = Ba, Sr, and Ca; *x* = 1) materials were constructed using 1 × 1 × 1 cells by replacing four of the twelve S atoms with O, corresponding to 33.33% S → O substitution. The same ordered S/O substitution motif was maintained across all three compositions. The corresponding initial oxygen fractional coordinates are compared in Table S9a of the SI. Full structural relaxation of the resulting ASnS_2_O structures produced low-symmetry triclinic configurations, which were used for the subsequent calculations.

To further investigate the influence of S/O ordering, 495 possible substitution patterns were considered for each BaSnS_3_- and SrSnS_3_-derived framework. Symmetry-based enumeration using pymatgen yielded 135 symmetry-distinct configurations per framework. These configurations were initially screened using the Forcite module with the Universal force field and Smart geometry optimization, while keeping the initial lattice parameters fixed. The five candidates with the lowest Forcite screening energies from each framework were then selected for CASTEP structural relaxation and DFT total-energy comparison. For CaSnS_2_O, five configurations were generated using the selected Sr-derived S/O ordering patterns and subsequently evaluated using CASTEP, without separate enumeration or Forcite screening. As only selected configurations were evaluated at the DFT level, the results do not establish global configurational ground states. Further details are provided in Section IX and Tables S9–S11 of the SI.

Exchange–correlation interactions were described using the Perdew–Burke–Ernzerhof generalized-gradient approximation (GGA-PBE)^39^ together with norm-conserving pseudopotentials. A plane-wave kinetic-energy cutoff of 650 eV and a 5 × 3 × 2 Monkhorst–Pack *k*-point mesh^40^ were employed. Convergence tests with respect to the *k*-point mesh and plane-wave cutoff were performed for BaSnS_2_O, and the corresponding results are provided in the SI (Table S4 and Fig. S1). The band-structure k-path was generated automatically based on the optimized crystal lattice. Structural relaxation was carried out using the Broyden–Fletcher–Goldfarb–Shanno (BFGS) minimization scheme. The self-consistent-field convergence tolerance was set to 1.0 × 10^−6^ eV atom^−1^. Geometry optimization was considered converged when the total-energy change, maximum ionic force, maximum atomic displacement, and maximum stress were below 1.0 × 10^−5^ eV atom^−1^, 0.03 eV Å^−1^, 0.001 Å, and 0.05 GPa, respectively. To investigate the band-gap evolution, CASTEP Mulliken bond populations were analyzed using 11 Sn–S and 5 Sn–O atom-pair entries per compound. These values represent total bond overlaps rather than orbital-resolved contributions. The highest-valence- and lowest-conduction-band widths were extracted along the sampled G–F–Q–Z–G path. Additional fixed-volume calculations were performed by compressing BaSnS_2_O to the CaSnS_2_O equilibrium volume and expanding CaSnS_2_O to the BaSnS_2_O equilibrium volume. Atomic positions were relaxed at fixed lattice parameters, followed by PBE band-gap calculations. Detailed results are provided in Tables S5 and S6 of the SI. Additional DFT+*U* (PBE+*U*) calculations were performed on PBE-optimized ASnS_2_O (A = Ba, Sr, Ca) structures by applying *U* = 2–4 eV to Sn-5p states without further geometry relaxation. The resulting band gaps were used for water-redox screening sensitivity analysis (Section VIII, Tables S7 and S8, SI).

The fully optimized structures were subsequently used to evaluate the structural parameters, electronic band structures, total and partial densities of states (DOS/PDOS), optical response, and elastic properties. The simulated XRD patterns were generated using Cu Kα radiation (*λ* ≈ 1.5406 Å) in Bragg–Brentano geometry over a 2*θ* range of 5°–60° with a step size of 0.05°. The optical properties were evaluated from the frequency-dependent complex dielectric response calculated using CASTEP. To account for the anisotropic optical behavior of the triclinic structures, polarization-dependent dielectric spectra were obtained along six directions: (1,0,0), (0,1,0), (0,0,1), (1,1,0), (1,0,1), and (0,1,1). The six independent components of the complex dielectric tensor were reconstructed from the directional responses using normalized Cartesian polarization vectors derived from the relaxed triclinic lattice, following *ε*_u_(*ω*) = *u*^*T*^*ε*(*ω*)*u*. For comparison among the three compositions, the polycrystalline-averaged dielectric function, absorption coefficient, refractive index, extinction coefficient, optical conductivity, reflectivity, and energy-loss function were obtained directly using the Polycrystalline option in CASTEP Analysis and analyzed over the photon-energy range of 0–35 eV. The reconstructed dielectric tensor components and the original (1,0,0)-polarized optical spectra are provided in the SI (Fig. S2–S9). The calculated optical spectra describe direct interband electronic transitions without explicitly including phonon-assisted indirect transitions. The 21 independent elastic stiffness constants were obtained by finite-strain calculations using four steps and a maximum strain amplitude of 0.003. To assess strain-amplitude sensitivity, additional elastic calculations were performed at maximum strain amplitudes of 0.002 and 0.004 using the same four-step procedure. The elastic constants were found to depend on the strain amplitude (Table S3 of the SI): the small off-diagonal constants showed the largest relative variations and occasional sign changes, and several diagonal constants (C_22_ of BaSnS_2_O and SrSnS_2_O, and C_11_ of CaSnS_2_O) also varied by more than 10 GPa. The values in Table 4 correspond to a maximum strain amplitude of 0.003; the derived polycrystalline properties are therefore regarded as indicative. Elastic stability was examined from the positive definiteness of the stiffness matrix,^41^ while the polycrystalline elastic moduli were evaluated using the Voigt–Reuss–Hill approximation.^42^ The Pugh ratio, Vickers hardness, universal elastic anisotropy index, and Debye temperature were also evaluated using established elastic models.^43–46^

The elemental and competing-phase reference structures were independently optimized using CASTEP (GGA-PBE) with norm-conserving pseudopotentials, a 650 eV plane-wave cutoff, and phase-specific Monkhorst–Pack *k*-point meshes. Their structural details, computational parameters, and reference energies are provided in Section XI and Table S13 of the SI.

The *Γ*-point phonon frequencies of the optimized ASnS_2_O structures were calculated using the density-functional perturbation theory (DFPT) linear-response method in CASTEP. Full phonon dispersions were not calculated.

The nominal water-redox band-edge positions were estimated by combining the CASTEP-calculated band-gap energies with the Mulliken electronegativity approach. The conduction-band minimum (CBM) and valence-band maximum (VBM) potentials were estimated using literature ionization-energy and electron-affinity data^47,48^ and referenced to the normal hydrogen electrode (NHE) scale. Their positions relative to the H+/H_2_ and O_2_/H_2_O redox levels at pH = 0 were used for qualitative screening of the water-redox band-edge alignment.

## 3. Results and discussion

### 3.1. Structural properties

Fig. 1 illustrates the fully optimized crystal structures of ASnS_2_O (A = Ba, Sr, and Ca), showing their atomic arrangements and elemental distributions. All quantitative structural parameters were extracted directly from the optimized CASTEP lattice vectors and fractional atomic coordinates.

**Fig. 1:**
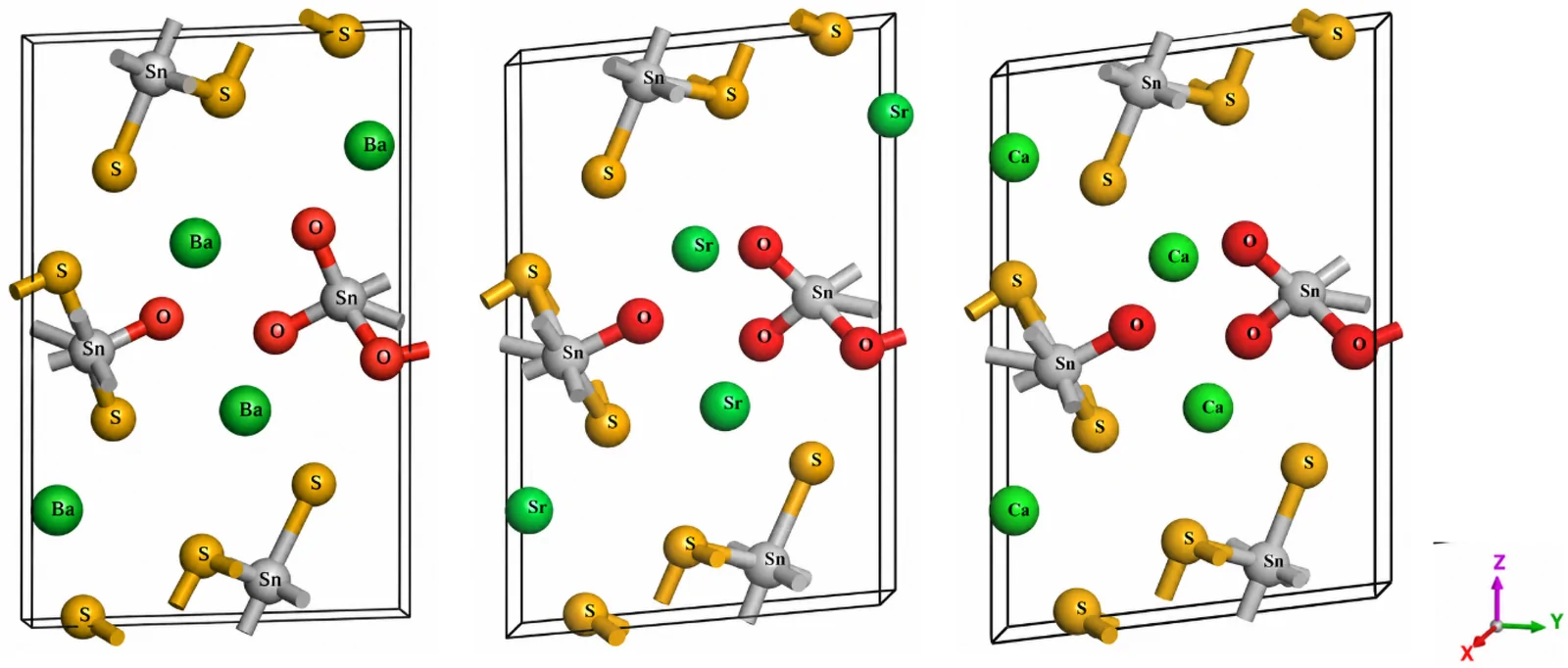
Fully optimized crystal structures of BaSnS_2_O (left), SrSnS_2_O (middle), and CaSnS_2_O (right).

For the local structural analysis, Sn–anion and A-site–anion distances were evaluated from the optimized triclinic cells under periodic boundary conditions (PBC). For atoms *i* and *j*, the shortest periodic distance was obtained as
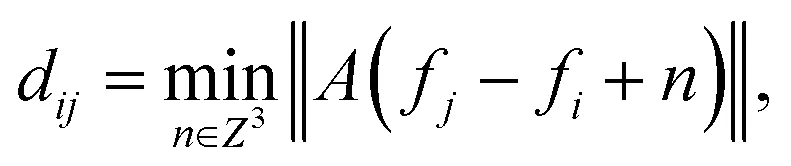
where *A* = [*a*, *b*, *c*] is the optimized lattice matrix, *f*_*i*_ and *f*_*j*_ are the fractional atomic coordinates, and *n* denotes integer lattice translations. Bond angles between two first-shell anions *j* and *k* around a central Sn atom *i* were determined from
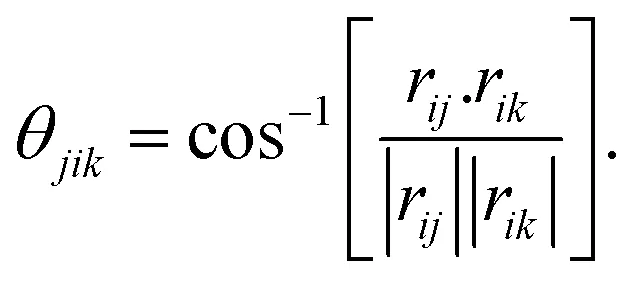


The first Sn–anion coordination shell was identified using a distance-gap criterion based on the sorted PBC-corrected Sn–O/S distances. The distinct separation between the first-shell and subsequent coordination contacts permits a common cutoff of 3.0 Å for all three compositions, consistent with the distance ranges reported in the footnote to Table 2. The distance calculations explicitly account for the non-orthogonal triclinic lattice and neighboring periodic images. The same PBC-corrected distance-sorting procedure was applied independently to each crystallographically inequivalent A site to identify the first A–O/S coordination shell from the separation between the near-neighbor group and the subsequent distance shell. The corresponding site-resolved coordination data are provided in Table S1 of the SI.

The final CASTEP total energies are included in Table 1 to provide energetic traceability for the fully optimized structures. Because BaSnS_2_O, SrSnS_2_O, and CaSnS_2_O contain different A-site species, these absolute total energies are not used for direct cross-composition stability comparisons.

**Table 1:** Optimized lattice parameters, space groups, unit-cell volumes, and total energies of ASnS_2_O (A = Ba, Sr, and Ca)

Compound	Space group	*a* (Å)	*b* (Å)	*c* (Å)	*α* (°)	*β* (°)	*γ* (°)	*V* (Å^3^)	Final total energy (eV per cell)
BaSnS_2_O	P1 (no. 1)	3.9036	8.4495	13.5326	88.011	90.935	90.685	446.001	−7127.991533
SrSnS_2_O	P1 (no. 1)	3.8002	8.4086	13.2043	83.083	90.782	89.569	418.804	−7703.562351
CaSnS_2_O	P1 (no. 1)	3.7006	8.4716	12.7294	81.186	90.451	89.771	394.341	−8339.507607

As summarized in Table 1, the optimized lattice shows a systematic contraction along the Ba → Sr → Ca sequence. The *a* and *c* parameters decrease progressively, whereas *b* varies only slightly, resulting in a reduction of the unit-cell volume from 446.001 Å^3^ for BaSnS_2_O to 394.341 Å^3^ for CaSnS_2_O, corresponding to approximately 11.6% contraction. This trend is consistent with the decreasing effective size of the alkaline-earth cations from Ba to Sr to Ca and can be interpreted as an increasing chemical-pressure effect on the host framework.^25^

Crystallographic symmetry analysis identified the initial S/O-ordered BaSnS_2_O, SrSnS_2_O, and CaSnS_2_O structures as monoclinic *Pm* (space group No. 6). Following full structural relaxation, all three compositions adopted the polar, non-centrosymmetric triclinic *P*1 space group (No. 1), rather than the centrosymmetric *P*1̄ space group. This relaxation-induced symmetry lowering is accompanied by pronounced anisotropic lattice distortion. In particular, α decreases from 88.011° for BaSnS_2_O to 81.186° for CaSnS_2_O, whereas *β* and *γ* remain relatively close to 90°. Thus, A-site variation induces not only a reduction in unit-cell volume but also a substantial angular rearrangement of the triclinic lattice. The unequal responses of *a*, *b*, *c*, and the lattice angles indicate that the structural relaxation is governed by directional deformation rather than simple isotropic compression.

PBC-corrected A-site–anion distance sorting further reveals strongly distorted and site-dependent A-site coordination environments in the relaxed P1 structures. The average A-site coordination numbers are 9.00, 8.75, and 8.75 for BaSnS_2_O, SrSnS_2_O, and CaSnS_2_O, respectively. Thus, the relaxed structures do not retain a single ideal A-site coordination polyhedron. The detailed site-resolved coordination numbers, first-shell distance ranges, and subsequent contacts are summarized in Table S1 of the SI.

For quantitative comparison of the A-site size effect, the eight-coordinate Shannon radii, *r*(Ba^2+^) = 1.42 Å, *r*(Sr^2+^) = 1.26 Å, and *r*(Ca^2+^) = 1.12 Å, were examined as a common effective size descriptor,^25^ rather than as evidence of exact eightfold coordination at every A site. The optimized cell volumes of 446.001, 418.804, and 394.341 Å^3^ yield volume–radius slopes of 170.0 Å^3^ Å^−1^ for Ba → Sr and 174.7 Å^3^ Å^−1^ for Sr → Ca, respectively. These slopes differ by only approximately 2.8%, and a linear fit gives *R*^2^ = 0.99994. By comparison, the CN = 6 and CN = 12 Shannon radius sets give substantially larger relative slope spreads of approximately 16% and 42%, respectively. Thus, among the Shannon radius sets examined, the CN = 8 values provide the most internally consistent common effective size scale for describing the Ba → Sr → Ca volume trend. The complete quantitative comparison is provided in Table S2 of the SI.

From BaSnS_2_O to CaSnS_2_O, the CN = 8 Shannon radius decreases by approximately 21.1%, whereas the unit-cell volume decreases by 11.6%, corresponding to an equivalent isotropic linear contraction of only approximately 4.0%. This disparity indicates that the A-site size variation is not accommodated through uniform lattice compression alone; instead, a substantial fraction of the size change is accommodated through anisotropic lattice deformation and local rearrangement of the Sn– (S, O) framework.

The local coordination analysis reveals that the Sn–O distances remain comparatively stable across the three compositions, whereas the Sn–S environment responds more strongly to A-site variation. In particular, the upper limit of the first-shell Sn–S distance decreases from 2.854 Å for BaSnS_2_O to 2.689 Å for CaSnS_2_O. Consequently, the Sn–S distance-distribution width narrows from 0.479 Å in BaSnS_2_O to 0.393 Å in SrSnS_2_O and 0.281 Å in CaSnS_2_O. This indicates a progressively narrower distribution of first-shell Sn–S coordination distances. Importantly, this geometrical trend alone does not establish stronger Sn–S bonding; complementary Mulliken overlap analysis is presented in Section 3.2.2 and Table S5 of the SI.

The bond-angle distributions provide further evidence of local structural reorganization. Broad S–Sn–S and O–Sn–S ranges indicate strongly distorted Sn-centered mixed-anion environments, while the O–Sn–O range evolves from 104.23–162.36° in BaSnS_2_O to 96.78–174.78° in CaSnS_2_O. The coexistence of markedly bent and nearly linear arrangements demonstrates that the macroscopic cell contraction is accommodated through substantial local angular relaxation rather than a uniform shortening of all Sn–anion contacts.

The contrasting Sn–S and Sn–O bond-length responses can be interpreted in terms of anisotropic structural relaxation and differences in their local bonding environments. From BaSnS_2_O to CaSnS_2_O, the mean Sn–S distance decreases slightly from 2.564 to 2.536 Å (Table S6), whereas the maximum first-shell Sn–S distance decreases from 2.854 to 2.689 Å (Table 2), indicating a more pronounced redistribution of longer Sn–S contacts. In contrast, Sn–O distances remain comparatively stable. The CASTEP Mulliken analysis (Table S5) shows that the mean Sn–O overlap populations vary only slightly (0.502–0.522), consistent with limited changes in Sn–O electronic overlap. These results suggest that A-site contraction is accommodated partly through Sn–S coordination rearrangements and local angular distortions, although Mulliken populations alone do not establish relative bond stiffness. The comparatively short Sn–O bonds are likely less compliant than the longer Sn–S contacts, which may account for the larger redistribution among the Sn–S distances; this interpretation is not directly established by the present calculations.

**Table 2:** First-shell Sn–anion bond-length and bond-angle ranges in the fully optimized ASnS_2_O (A = Ba, Sr, and Ca) structures^a^

Compound	Sn–O (Å)	Sn–S (Å)	S–Sn–S (°)	O–Sn–S (°)	O–Sn–O (°)
BaSnS_2_O	1.823–1.889	2.375–2.854	80.54–177.31	77.52–130.55	104.23–162.36
SrSnS_2_O	1.816–1.880	2.395–2.788	84.02–179.61	78.84–130.66	99.66–169.41
CaSnS_2_O	1.815–1.915	2.408–2.689	85.97–177.95	80.06–133.59	96.78–174.78

a The first Sn–anion coordination shell was identified from the separation in the sorted PBC-corrected Sn–O/S distance distributions. The largest first-shell distances are 2.854, 2.788, and 2.689 Å for BaSnS_2_O, SrSnS_2_O, and CaSnS_2_O, respectively, whereas the subsequent contacts occur at 3.347, 3.297, and 3.211 Å. The reported values represent the minimum–maximum ranges over all Sn sites.

Overall, the optimized structures exhibit a clear Ba → Sr → Ca reduction in unit-cell volume accompanied by anisotropic lattice distortion and redistribution of the local Sn–S/O coordination geometry. These structural modifications alter the geometrical environment governing Sn–anion orbital interactions and provide a physical basis for interpreting the composition-dependent electronic and optical responses of the ASnS_2_O series.

#### 3.1.1. Simulated X-ray diffraction analysis

The simulated X-ray diffraction (XRD) patterns of BaSnS_2_O, SrSnS_2_O, and CaSnS_2_O obtained from the fully optimized structures are presented in Fig. 2. Selected principal reflections were indexed against the corresponding optimized unit cells to provide a direct crystallographic interpretation of the calculated diffraction features. The three compounds exhibit clear composition-dependent changes in both peak positions and relative intensities.

**Fig. 2:**
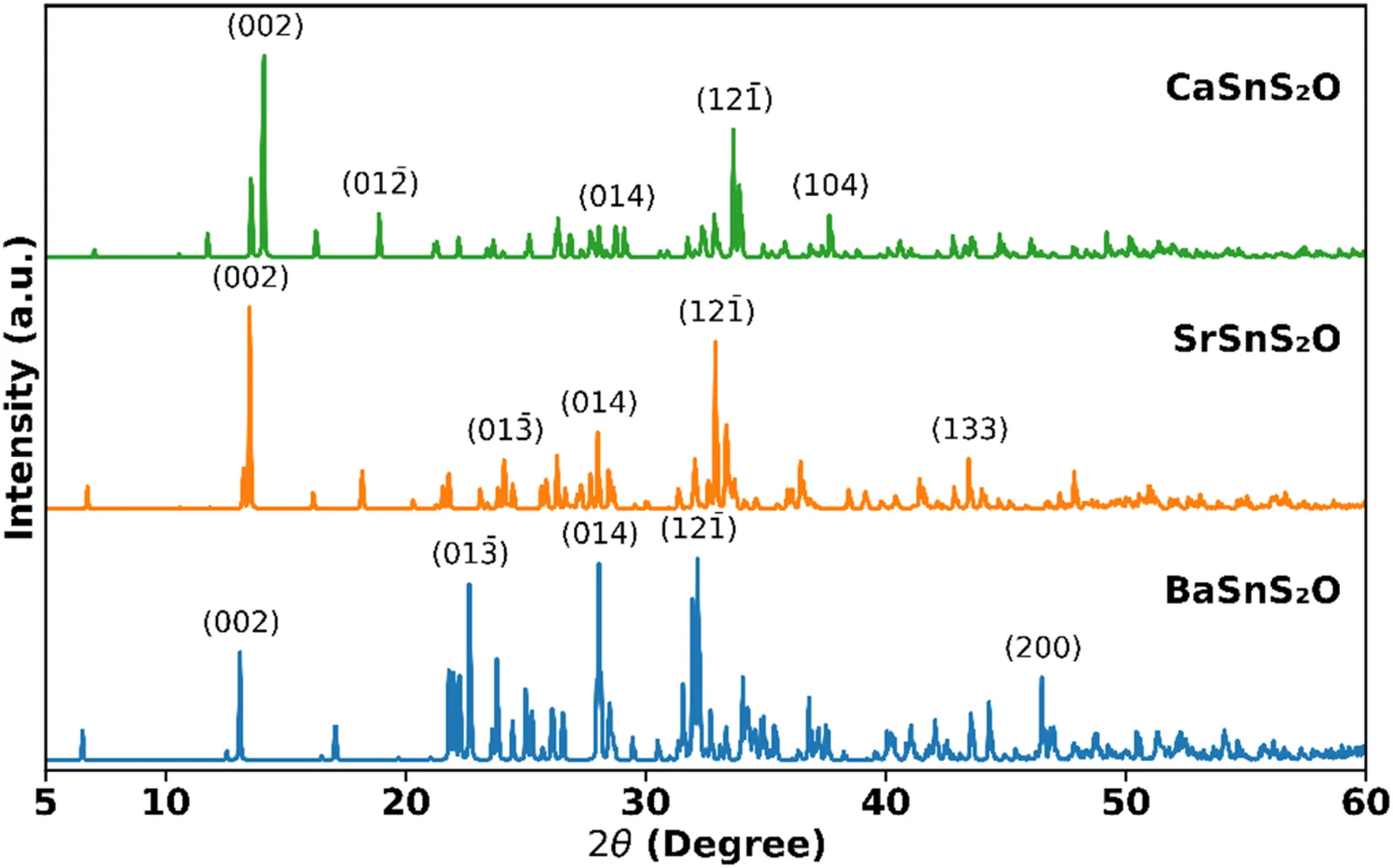
Simulated X-ray diffraction patterns of BaSnS_2_O, SrSnS_2_O, and CaSnS_2_O calculated from the fully optimized structures using Cu Kα radiation. Selected principal reflections are indexed according to the corresponding optimized unit cells.

A systematic shift of the low-angle (002) reflection is observed from approximately 13.10° for BaSnS_2_O to 13.50° for SrSnS_2_O and 14.10° for CaSnS_2_O. According to Bragg's relation, 2*d* sin *θ* = *nλ*, the shift toward higher 2*θ* indicates a decrease in the corresponding interplanar spacing and is consistent with the calculated Ba → Sr → Ca lattice contraction.^49^ Similar composition-dependent changes are evident for other indexed reflections, including (014) and (121̄). The peak shifts are not uniform across all reflections, consistent with the anisotropic changes in the lattice parameters and triclinic angles established from the optimized structures.

The redistribution of relative peak intensities reflects changes in the calculated structure factors associated with A-site substitution and the accompanying relaxation of the atomic coordinates. The comparatively dense diffraction patterns are also compatible with the low-symmetry triclinic P1 structures obtained after relaxation. Overall, the indexed XRD patterns provide a reciprocal-space representation of the composition-dependent lattice contraction and anisotropic structural reorganization across the ASnS_2_O series.

### 3.2. Electronic properties

#### 3.2.1. Electronic band structure

The calculated electronic band structures of CaSnS_2_O, SrSnS_2_O, and BaSnS_2_O along the automatically generated G–F–Q–Z–G k-path are presented in Fig. 3. All three compounds exhibit semiconducting behavior with indirect band gaps in the range of 1.461–1.928 eV. For CaSnS_2_O [Fig. 3(a)], the VBM occurs at *G*, whereas the CBM is located within the G–F segment, resulting in an indirect band gap of 1.928 eV. In SrSnS_2_O [Fig. 3(b)] and BaSnS_2_O [Fig. 3(c)], the VBM is located at *Z* while the CBM occurs at F, giving indirect gaps of 1.727 and 1.461 eV, respectively. Thus, the VBM and CBM occur at different wave vectors in each compound, indicating that the lowest-energy inter-band excitation involves a momentum change rather than a direct vertical transition in reciprocal space.

**Fig. 3:**
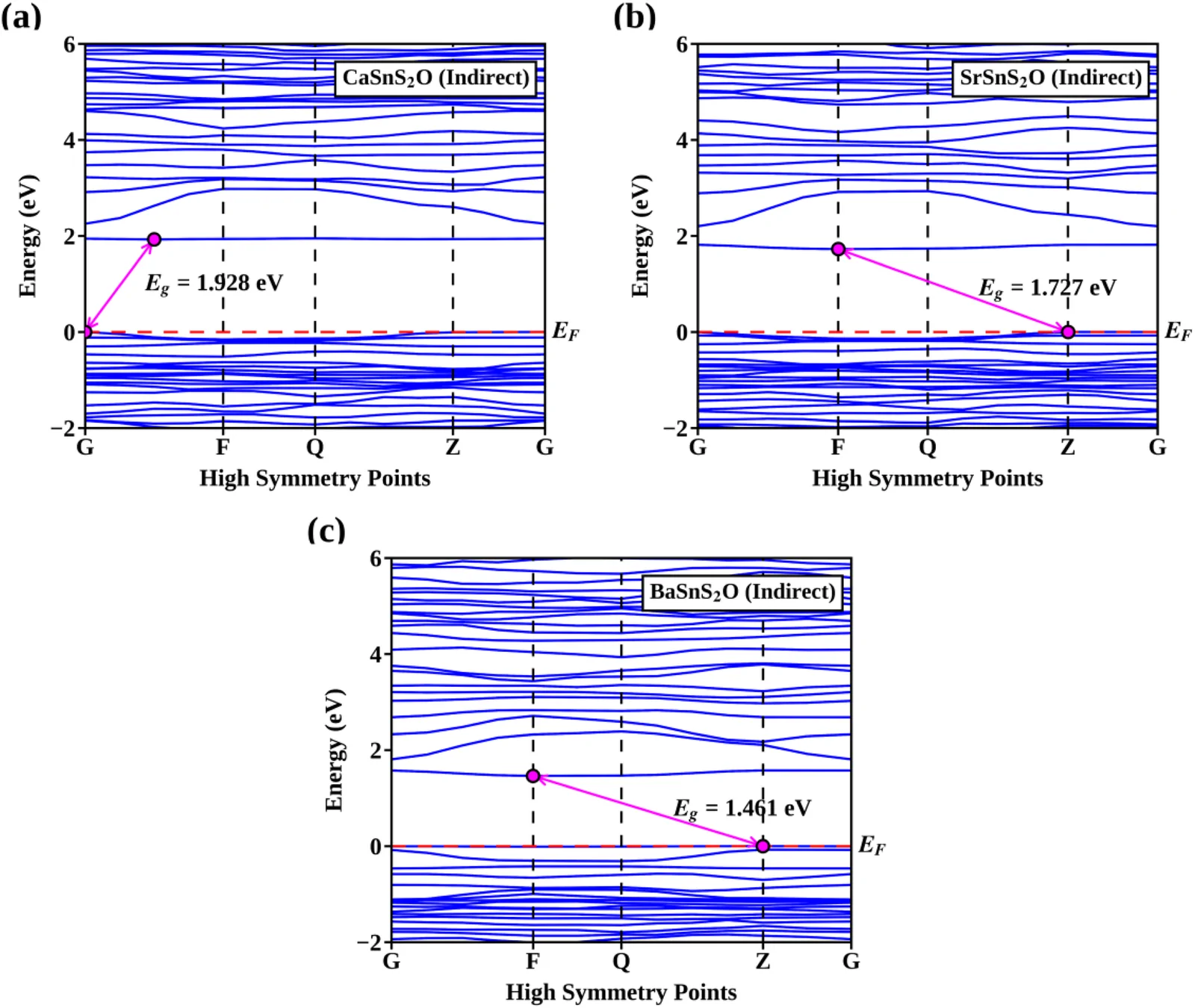
Calculated electronic band structures of the primary configurations of (a) CaSnS_2_O, (b) SrSnS_2_O, and (c) BaSnS_2_O along the automatically generated G–F–Q–Z–G k-path. The Fermi level is set to 0 eV, and the positions of the VBM and CBM together with the calculated indirect band gaps are indicated.

For the common S/O substitution motif adopted in the primary calculations, a systematic compositional evolution is observed, following *E*_g_(BaSnS_2_O) < *E*_g_(SrSnS_2_O) < *E*_g_(CaSnS_2_O), with the band gap increasing from 1.461 to 1.727 and finally 1.928 eV. This evolution accompanies the Ba → Sr → Ca structural contraction established above, where the optimized unit-cell volume decreases from 446.001 to 394.341 Å^3^, together with substantial anisotropic lattice deformation and rearrangement of the local Sn–S/O coordination. Such changes in bond geometry and interatomic separation can modify orbital overlap and the relative energies and dispersions of the band-edge states. Therefore, the observed gap evolution is physically consistent with a composition-dependent reorganization of the electronic structure. Nevertheless, the correlation should not be interpreted as arising from volume contraction alone, because changes in mixed-anion coordination and local distortion occur simultaneously. Because GGA-PBE can underestimate semiconductor band gaps, the calculated *E*_g_ values are used primarily to examine the relative electronic trend across the ASnS_2_O series within the common S/O substitution motif rather than as quantitatively exact gap predictions. To evaluate the sensitivity of the calculated band gaps, additional Sn-5p PBE+*U* calculations were performed with *U* = 2–4 eV. The resulting band gaps increase to 1.497–1.537 eV for BaSnS_2_O, 1.788–1.854 eV for SrSnS_2_O, and 1.992–2.058 eV for CaSnS_2_O, while the ordering *E*_g_(BaSnS_2_O) <*E*_g_(SrSnS_2_O) <*E*_g_(CaSnS_2_O) remains unchanged (Table S7, SI). These results demonstrate that the relative band-gap ordering is preserved within the investigated *U* range, although the absolute band-gap accuracy remains unverified.

To assess the influence of S/O anion ordering on the electronic properties, five selected configurations for each ASnS_2_O composition were further investigated using CASTEP. The calculated band gaps span 0.920–1.865 eV for BaSnS_2_O, 1.405–2.099 eV for SrSnS_2_O, and 1.397–2.026 eV for CaSnS_2_O. Relative to the lowest DFT total energy within each evaluated five-configuration subset, the energy differences span 0–3.2509 eV per cell for BaSnS_2_O, 0–0.6297 eV per cell for SrSnS_2_O, and 0–0.7416 eV per cell for CaSnS_2_O, indicating appreciable energetic differences among the evaluated configurations. These results highlight the sensitivity of both the electronic band gaps and relative configurational energetics to S/O ordering. Accordingly, the Ba–Sr–Ca band-gap trend obtained for the primary structures should be interpreted within the common S/O substitution motif adopted for the comparative analysis. Nevertheless, because DFT calculations were restricted to selected configurations, the present results do not establish the global configurational ground states (Table S11, SI). For reference, the primary BaSnS_2_O structure used in the comparative analysis (−7127.99 eV per cell, Table 1) lies 1.31 eV per cell above the lowest-energy BaSnS_2_O candidate among those evaluated (−7129.30 eV per cell, Table S11), and the primary CaSnS_2_O structure (−8339.51 eV per cell) lies 0.92 eV per cell above the lowest-energy CaSnS_2_O candidate (−8340.42 eV per cell), whereas the primary SrSnS_2_O structure (−7703.56 eV per cell) lies only 0.02 eV per cell above its lowest-energy candidate (−7703.58 eV per cell). The primary configurations were adopted because they share a common S/O motif across the series; they should therefore not be regarded as ground-state configurations, and the absolute band gaps may differ for lower-energy arrangements.

To quantify the band-edge dispersion observed in Fig. 3, the bandwidths of the highest valence band and lowest conduction band were evaluated along the sampled G–F–Q–Z–G k-path. The highest-valence-band widths are 0.011, 0.142, and 0.157 eV for BaSnS_2_O, SrSnS_2_O, and CaSnS_2_O, respectively, whereas the corresponding lowest-conduction-band widths are 0.115, 0.090, and 0.020 eV. These results reveal contrasting dispersion trends across the Ba → Sr → Ca series: the highest valence band becomes more dispersive, while the lowest conduction band becomes progressively flatter. These bandwidths describe the sampled k-path rather than the entire Brillouin zone. Directional effective masses were obtained from local parabolic fits to the band-edge dispersions using m* = ℏ^2^/(*d*^2^*E*/*dk*^2^), and the calculated values are summarized in Table 3.

**Table 3:** Highest-valence-band and lowest-conduction-band widths and directional carrier effective masses of ASnS_2_O (A = Ba, Sr, and Ca)

Compound	Highest VB width (eV)	Lowest CB width (eV)	Carrier	Direction	Effective mass, m*/*m*_0_
CaSnS_2_O	0.157	0.020	Electron	G–F, around the off-symmetry CBM	6.77
			Hole	G–F	1.15
			Hole	Z–G	22.43
SrSnS_2_O	0.142	0.090	Electron	G–F	4.30
			Electron	F–Q	13.35
			Hole	Q–Z	1.45
			Hole	Z–G	122.76
BaSnS_2_O	0.011	0.115	Electron	G–F	2.68
			Electron	F–Q	26.46
			Hole	Q–Z	39.12
			Hole	Z–G	50.19

For CaSnS_2_O, the CBM lies within the G–F segment rather than at a high-symmetry point; therefore, only the electron effective mass along the sampled G–F direction is reported. The generally large and strongly direction-dependent effective masses, particularly along the weakly dispersive directions, are consistent with the flat band-edge features evident in Fig. 3 and suggest less favorable band-like carrier transport along these directions.

#### 3.2.2. Density of states and orbital contributions

The total and orbital-projected densities of states (DOS/PDOS) of CaSnS_2_O, SrSnS_2_O, and BaSnS_2_O are shown in Fig. 4(a–c), respectively, with the Fermi level set to 0 eV. In all three compounds, the absence of electronic states around the Fermi level confirms their semiconducting character, consistent with the corresponding band structures. The evolution of the DOS gap also follows the calculated indirect band-gap sequence of 1.461, 1.727, and 1.928 eV for BaSnS_2_O, SrSnS_2_O, and CaSnS_2_O, respectively.

**Fig. 4:**
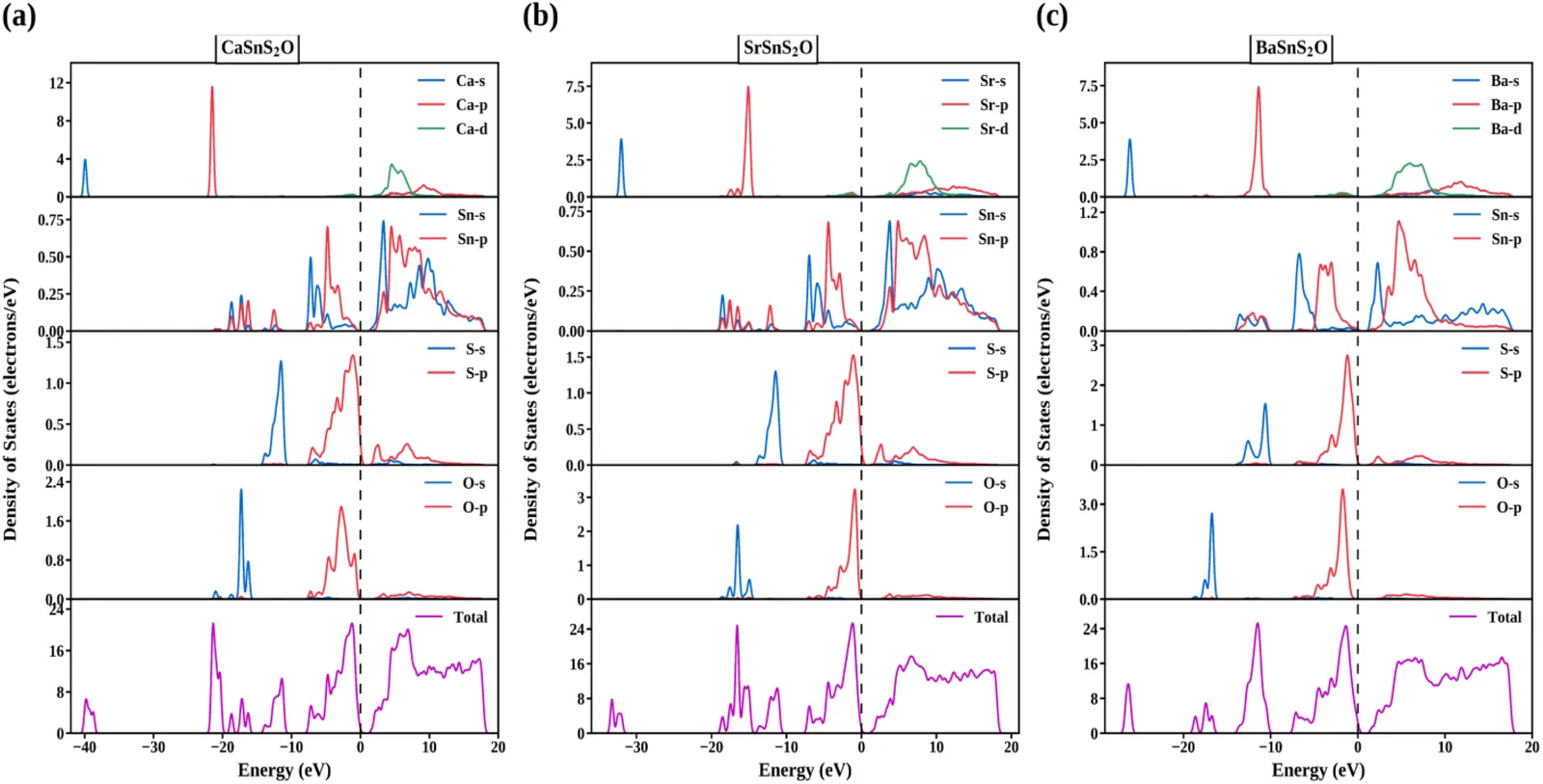
Total and orbital-projected densities of states of (a) CaSnS_2_O, (b) SrSnS_2_O, and (c) BaSnS_2_O. The vertical dashed line indicates the Fermi level at 0 eV.

The PDOS profiles reveal that the upper valence region is dominated mainly by anion p states. S–p orbitals provide substantial spectral weight close to the VBM, while O–p states contribute strongly within the occupied region immediately below the band edge. Sn–s/p states overlap with these anion-derived states at several energies, indicating appreciable orbital mixing within the Sn– (S, O) framework. In contrast, the S–s and O–s states occur considerably deeper in the valence region and therefore make little direct contribution to the frontier states.

Immediately above the band gap, the conduction-band states are predominantly Sn-derived, mainly involving Sn–s/p contributions with smaller anion-p components. The Ba, Sr, and Ca orbitals contribute only weakly at the band edges; their more pronounced d-state contributions appear several electron volts above the CBM. Thus, the electronic gap is governed primarily by the Sn– (S, O) sublattice rather than by direct A-site electronic states.

The pronounced Sn-derived spectral weight near the conduction edge is consistent with the weak dispersion of the lowest conduction band identified from the band-structure analysis. A plausible contribution to this behavior is the strongly distorted mixed-anion Sn–S/O coordination environment quantified in Section 3.1 and Table 2. The first-shell Sn–S distances span 2.375–2.854 Å in BaSnS_2_O, 2.395–2.788 Å in SrSnS_2_O, and 2.408–2.689 Å in CaSnS_2_O, while the broad Sn-centered bond-angle distributions further indicate substantial local distortion. Such distortion may reduce effective Sn–anion orbital overlap and intersite coupling along particular directions, thereby contributing to the narrow dispersion of the Sn-derived conduction-edge state. Although additional S/O configurations were assessed for energy and band-gap sensitivity, detailed band-structure and orbital analyses were limited to the primary configurations. Thus, the ordering dependence of the unusually flat conduction band remains unresolved.

To clarify the composition-dependent band-gap evolution, the effects of local Sn–anion bonding, band-edge dispersion, and lattice-volume variation were examined together. Although the equilibrium unit-cell volume decreases from 446.001 Å^3^ for BaSnS_2_O to 394.341 Å^3^ for CaSnS_2_O, accompanied by a reduction in the upper limit of the first-shell Sn–S distance distribution from 2.854 to 2.689 Å (Tables 1 and 2), the band gap increases from 1.461 to 1.928 eV. However, the CASTEP Mulliken analysis does not reveal a corresponding monotonic increase in Sn–S bond overlap. The mean Sn–S overlap populations are 0.655, 0.639, and 0.640 for BaSnS_2_O, SrSnS_2_O, and CaSnS_2_O, respectively, while the corresponding Sn–O populations are 0.502, 0.522, and 0.512 (Table S5, SI). These results do not support a simple explanation based solely on Sn–S bond shortening and increased bond overlap. Furthermore, the sampled-path width of the highest valence band increases from 0.011 to 0.142 and 0.157 eV across the Ba → Sr → Ca series, whereas the corresponding lowest-conduction-band width decreases from 0.115 to 0.090 and 0.020 eV (Table 3). This contrasting behavior indicates different changes in the dispersion of the valence- and conduction-edge states. To further examine the role of lattice volume, reciprocal volume-constrained calculations were performed. Compressing BaSnS_2_O to approximately the equilibrium volume of CaSnS_2_O reduces its band gap from 1.461 to 1.389 eV, whereas expanding CaSnS_2_O to approximately the equilibrium volume of BaSnS_2_O reduces its band gap from 1.928 to 1.795 eV (Table S6, SI). At nearly identical volumes, the Ca- and Ba-containing systems retain band-gap differences of 0.539 and 0.334 eV at the Ca- and Ba-equilibrium volumes, respectively. These findings show that the observed band-gap widening cannot be explained by unit-cell contraction alone and is consistent with the combined influence of A-site composition, local Sn–S/O coordination, and band-edge electronic reorganization. Nevertheless, the present Mulliken populations describe total atom-pair overlaps rather than orbital-pair-resolved interactions, while the volume-constrained comparisons do not uniquely separate compositional effects from changes in lattice shape and relaxed local geometry.

For comparison, previously reported ASnS_3_ phases have been shown to exhibit predominantly S-3p-derived valence states and Sn/S-derived conduction states.^18^ The present mixed-anion systems retain this general frontier-state framework while introducing additional O-2p contributions and considerable structural reconstruction. This comparison is qualitative because the previously reported ASnS_3_ and present mixed-anion systems differ in both composition and computational treatment. Finally, the anion-p and Sn-derived band-edge states identify the principal orbital manifolds relevant to low-energy optical transitions, although their transition probabilities must be established from the calculated optical response rather than from DOS alone.

### 3.3. Optical properties

The optical response of ASnS_2_O (A = Ba, Sr, and Ca) was investigated through the dielectric function, absorption coefficient, refractive index, extinction coefficient, optical conductivity, reflectivity, and energy-loss function. Owing to their triclinic crystal symmetry, these compounds exhibit direction-dependent optical responses that cannot be fully characterized by a single polarization direction. Therefore, the present analysis focuses on the polycrystalline-averaged optical spectra to provide an orientation-averaged comparison across the three compositions. The corresponding frequency-dependent complex dielectric tensor components and the previously calculated (1,0,0)-polarized optical spectra are presented in the SI (Fig. S2–S9) to document the directional optical response.

#### 3.3.1. Dielectric function

The calculated polycrystalline-averaged real, *ε*_1_(*ω*), and imaginary, *ε*_2_(*ω*), components of the dielectric function for BaSnS_2_O, SrSnS_2_O, and CaSnS_2_O are presented in Fig. 5(a and b). The real component describes the dispersive electronic polarization response, whereas the imaginary component represents the absorptive contribution arising from optically allowed interband electronic transitions.^50^ Because these compounds crystallize in the triclinic system, their dielectric responses are inherently direction-dependent. The polycrystalline-averaged spectra are therefore used to compare their orientation-averaged optical behavior, while the corresponding frequency-dependent dielectric tensor components are provided in the SI (Fig. S2–S4).

**Fig. 5:**
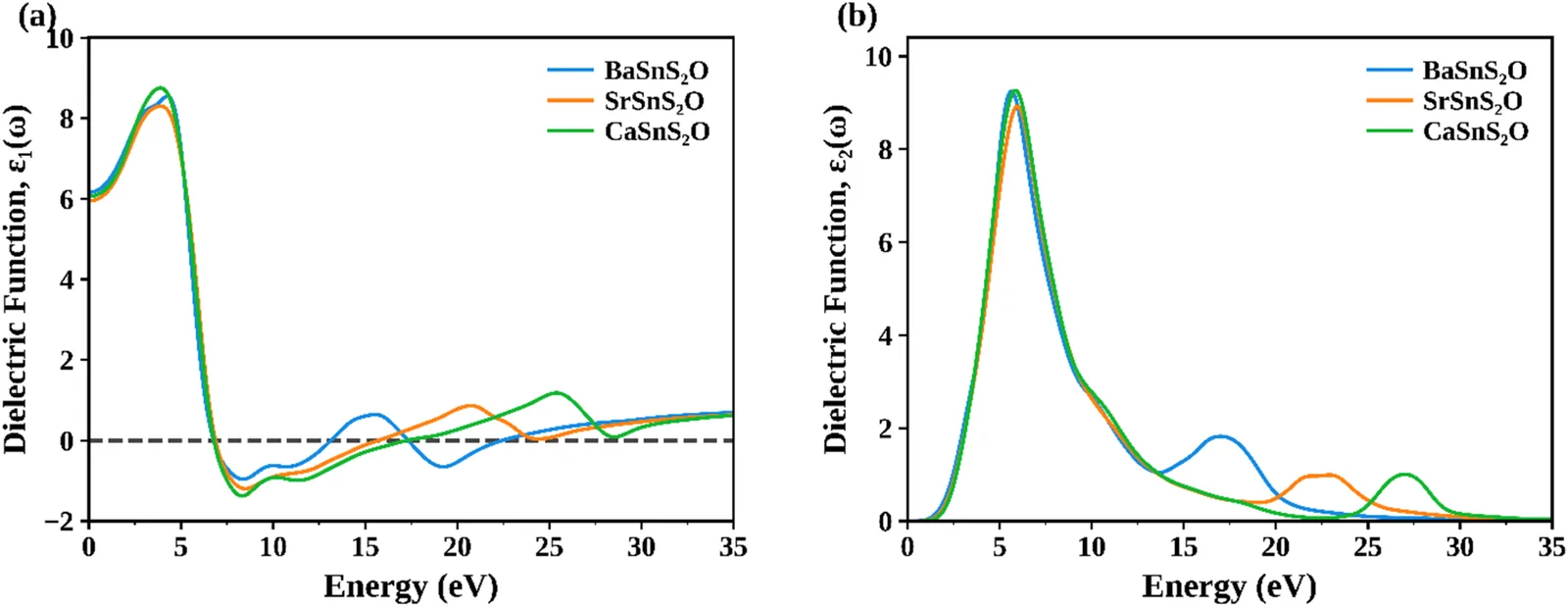
Polycrystalline-averaged (a) real *ε*_1_(*ω*) and (b) imaginary *ε*_2_(*ω*) dielectric functions of BaSnS_2_O, SrSnS_2_O, and CaSnS_2_O over 0–35 eV. The dashed line in (a) marks *ε*_1_(*ω*) = 0.

As illustrated in Fig. 5(a), the three compounds exhibit comparable low-energy dielectric responses, with *ε*_1_ approaching approximately 6.16, 5.95, and 6.06 for BaSnS_2_O, SrSnS_2_O, and CaSnS_2_O, respectively. With increasing photon energy, *ε*_1_ rises sharply and reaches prominent maxima of approximately 8.3–8.8 near 4 eV. Among the investigated compounds, CaSnS_2_O exhibits the highest maximum, followed by BaSnS_2_O and SrSnS_2_O. Beyond these peaks, *ε*_1_ decreases rapidly and crosses zero at approximately 7 eV, becoming negative over an intermediate energy range. This behavior indicates pronounced dielectric dispersion associated with the energy-dependent electronic polarization response. However, negative *ε*_1_ values alone do not establish metallic or plasmonic behavior. At higher photon energies, weaker dispersive features appear around 15–17 eV for BaSnS_2_O, 20–22 eV for SrSnS_2_O, and 25–27 eV for CaSnS_2_O, revealing composition-dependent variations in the high-energy dielectric response.

The imaginary component, *ε*_2_(*ω*), shown in Fig. 5(b), exhibits intense principal peaks at approximately 5–6 eV for all three compounds, indicating substantial contributions from optically allowed interband electronic transitions. BaSnS_2_O and CaSnS_2_O display comparable peak intensities of approximately 9.2–9.3, whereas SrSnS_2_O exhibits a slightly lower maximum of about 8.9. Following these principal peaks, *ε*_2_ decreases considerably, although weaker secondary features remain distinguishable at higher photon energies. Specifically, BaSnS_2_O exhibits a secondary feature near 17 eV, SrSnS_2_O around 22–23 eV, and CaSnS_2_O near 27 eV. The progressive shift of these secondary features toward higher photon energies across the Ba → Sr → Ca series demonstrates a composition-dependent redistribution of the high-energy dielectric response. However, assigning individual spectral features to specific electronic transitions would require a detailed transition-resolved analysis. Beyond these secondary features, *ε*_2_ gradually approaches zero, indicating a diminishing contribution from optically allowed electronic excitations at higher photon energies.

#### 3.3.2. Absorption coefficient

The calculated polycrystalline-averaged absorption coefficients, *α*(*ω*), of BaSnS_2_O, SrSnS_2_O, and CaSnS_2_O are presented in Fig. 6 over the photon-energy range of 0–35 eV. All three compounds exhibit relatively weak absorption at low photon energies, followed by a pronounced increase with increasing photon energy. Broad absorption features develop within approximately 7–12 eV, where the absorption coefficients reach values on the order of 10^5^ cm^−1^. Although the three compounds exhibit broadly comparable behavior in this region, their absorption intensities and spectral distributions differ with A-site composition.

**Fig. 6:**
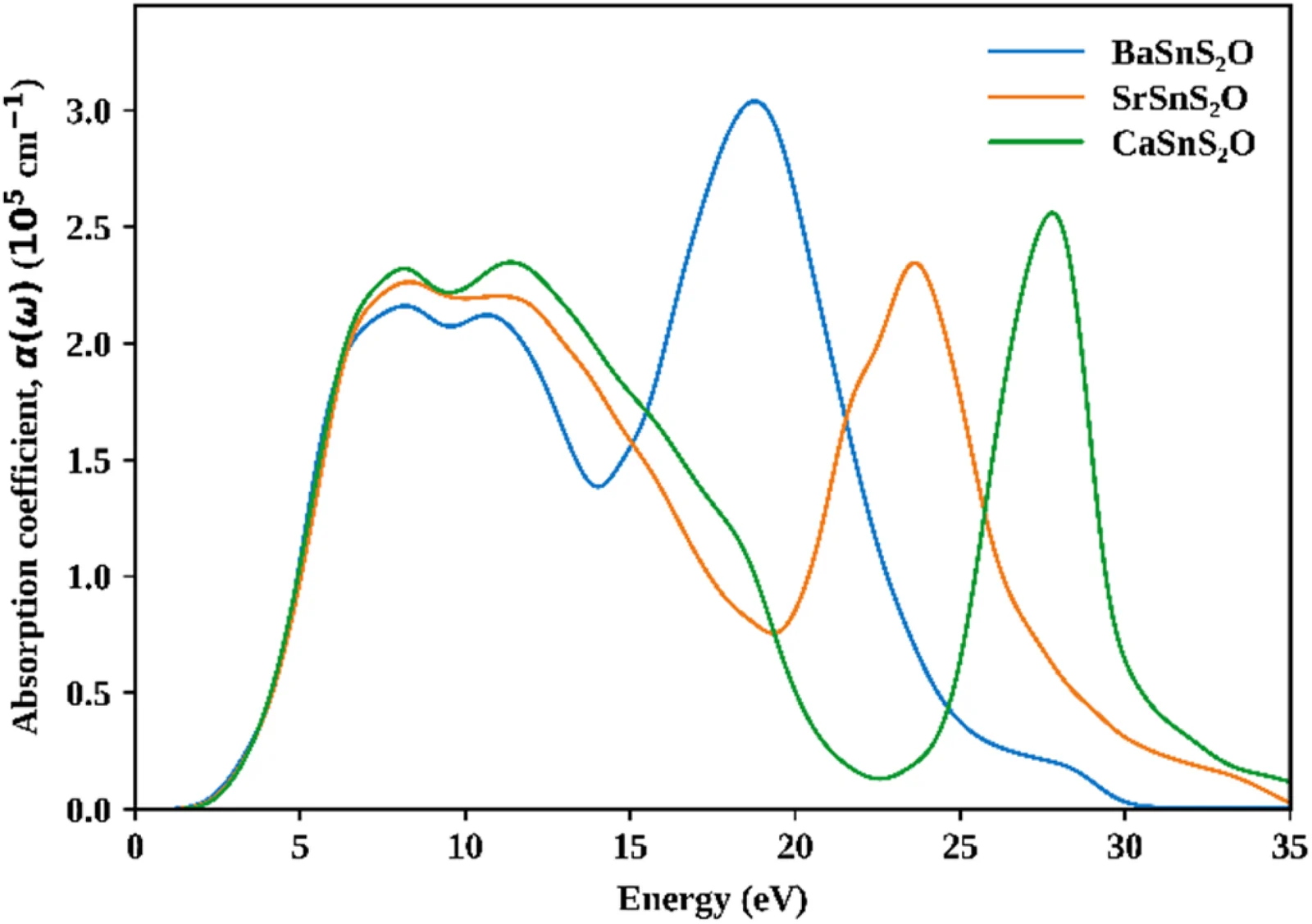
Polycrystalline-averaged absorption coefficients, *α*(*ω*), of BaSnS_2_O, SrSnS_2_O, and CaSnS_2_O over the photon-energy range of 0–35 eV.

More pronounced differences appear at higher photon energies. BaSnS_2_O exhibits the strongest absorption maximum of approximately 3.04 × 10^5^ cm^−1^ near 18.8 eV. In contrast, SrSnS_2_O reaches a maximum of approximately 2.35 × 10^5^ cm^−1^ near 23.6 eV, while CaSnS_2_O displays a pronounced maximum of approximately 2.56 × 10^5^ cm^−1^ near 27.8 eV. The positions of these high-energy absorption maxima shift progressively toward higher photon energies along the Ba → Sr → Ca series, whereas their intensities follow a non-monotonic trend. Beyond its dominant maximum, BaSnS_2_O shows a rapid reduction in absorption and becomes nearly negligible above approximately 30 eV. SrSnS_2_O and particularly CaSnS_2_O retain absorption contributions over a more extended high-energy range. These results demonstrate a clear composition-dependent redistribution of the calculated absorption response.

It is important to distinguish the calculated optical absorption from the fundamental electronic band gaps. The present optical spectra describe direct, vertical interband transitions between electronic states at the same crystal momentum and do not explicitly include phonon-assisted indirect transitions. However, BaSnS_2_O, SrSnS_2_O, and CaSnS_2_O possess indirect fundamental band gaps of 1.461, 1.727, and 1.928 eV, respectively. Because their valence-band maxima and conduction-band minima occur at different *k*-points, optical transitions across the fundamental indirect gaps require phonon assistance to satisfy momentum conservation. Consequently, the calculated absorption spectra do not capture these indirect transitions, and their direct-transition absorption onsets should not be identified with the fundamental indirect band-gap energies. Phonon-assisted processes may contribute to the experimental absorption onset but are beyond the scope of the present optical calculations.

The extended 0–35 eV energy range is considered to characterize the broader calculated interband optical response and resolve the composition-dependent high-energy absorption features. However, the dominant maxima observed at approximately 18–28 eV lie well beyond the visible and near-ultraviolet regions relevant to conventional solar-energy harvesting. Therefore, these high-energy absorption maxima should not be interpreted as direct evidence of efficient solar-spectrum absorption.

#### 3.3.3. Refractive index and extinction coefficient

The calculated polycrystalline-averaged refractive index, *n*(*ω*), and extinction coefficient, *k*(*ω*), of BaSnS_2_O, SrSnS_2_O, and CaSnS_2_O are presented in Fig. 7(a and b). The refractive index describes the dispersive optical response, whereas the extinction coefficient characterizes the attenuation of electromagnetic radiation within the material. As shown in Fig. 7(a), the three compounds exhibit comparable low-energy refractive indices of approximately 2.48, 2.44, and 2.46 for BaSnS_2_O, SrSnS_2_O, and CaSnS_2_O, respectively. With increasing photon energy, *n*(*ω*) rises sharply and reaches prominent maxima of approximately 3.0–3.1 near 4–5 eV. The closely grouped peaks indicate relatively small differences in the refractive response among the three compositions within this energy region.

**Fig. 7:**
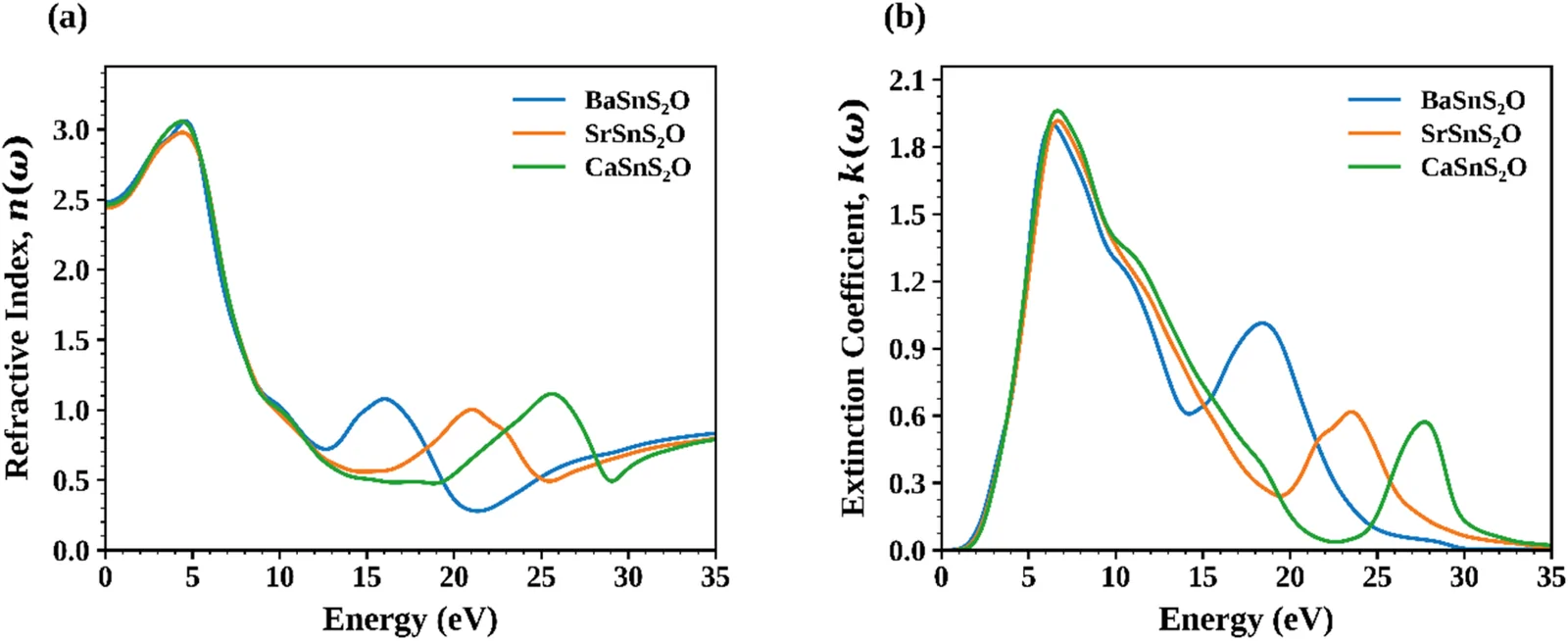
Polycrystalline-averaged (a) refractive index, *n*(*ω*), and (b) extinction coefficient, *k*(*ω*), of BaSnS_2_O, SrSnS_2_O, and CaSnS_2_O over 0–35 eV.

Beyond the principal maxima, *n*(*ω*) decreases rapidly and develops distinct composition-dependent dispersive features at higher photon energies. BaSnS_2_O exhibits a secondary maximum of approximately 1.1 near 16 eV, while SrSnS_2_O displays a corresponding feature of about 1.0 near 21 eV. For CaSnS_2_O, the secondary maximum shifts toward approximately 25–26 eV, reaching nearly 1.1. These variations reveal a progressive displacement of the higher-energy refractive features along the Ba → Sr → Ca series. Thus, although the low-energy refractive responses remain comparable, A-site substitution produces more pronounced differences in the higher-energy dispersion characteristics.

The extinction coefficient spectra in Fig. 7(b) exhibit intense principal peaks near 6–7 eV, with maximum values of approximately 1.9–2.0 for all three compounds. CaSnS_2_O displays the highest peak, followed closely by SrSnS_2_O and BaSnS_2_O. Beyond these maxima, *k*(*ω*) decreases considerably but exhibits additional composition-dependent features. BaSnS_2_O develops a pronounced secondary peak near 18–19 eV, reaching approximately 1.0, whereas SrSnS_2_O and CaSnS_2_O exhibit weaker secondary maxima of approximately 0.6 near 23–24 eV and 27–28 eV, respectively. The progressive displacement of these features toward higher photon energies demonstrates a composition-dependent redistribution of the optical attenuation response. At higher energies, *k*(*ω*) gradually approaches zero for all three compounds, indicating reduced optical attenuation within the upper part of the investigated energy range.

#### 3.3.4. Optical conductivity and reflectivity

The calculated polycrystalline-averaged real and imaginary components of the optical conductivity, *σ*_1_(*ω*) and *σ*_2_(*ω*), together with the reflectivity, *R*(*ω*), of BaSnS_2_O, SrSnS_2_O, and CaSnS_2_O are presented in Fig. 8(a–c). The real component, *σ*_1_(*ω*), represents the dissipative optical response, whereas the imaginary component, *σ*_2_(*ω*), describes the reactive response to the applied electromagnetic field. As shown in Fig. 8(a), *σ*_1_(*ω*) is negligible at low photon energies and increases sharply to prominent maxima of approximately 6.4–6.8 fs^−1^ near 6–7 eV. At higher energies, distinct secondary maxima appear around 17, 23, and 27 eV for BaSnS_2_O, SrSnS_2_O, and CaSnS_2_O, reaching approximately 3.8, 2.8, and 3.3 fs^−1^, respectively. These features reveal a progressive shift toward higher photon energies along the Ba → Sr → Ca series, while their intensities exhibit a non-monotonic variation.

**Fig. 8:**
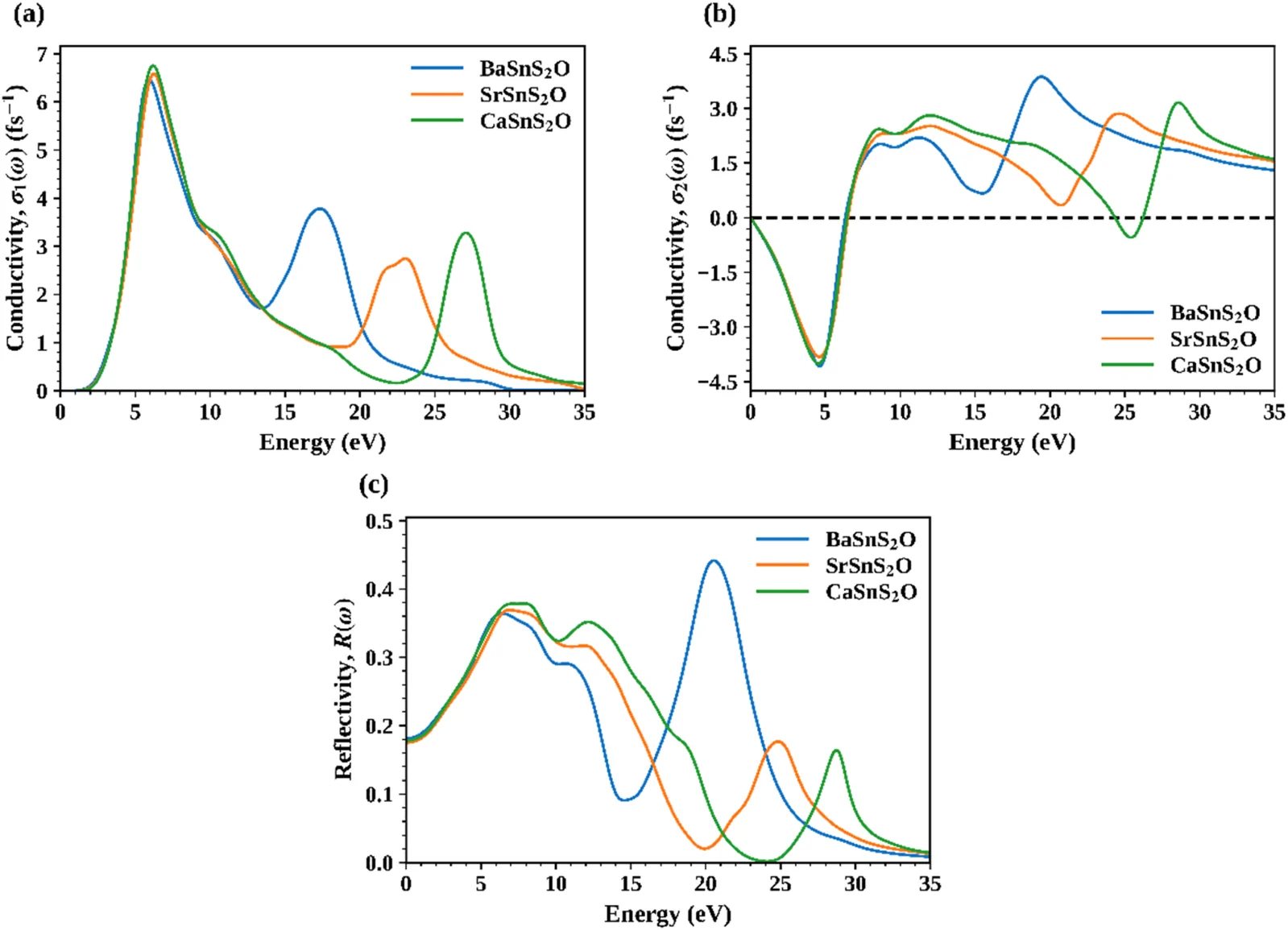
Polycrystalline-averaged (a) real optical conductivity *σ*_1_(*ω*), (b) imaginary optical conductivity *σ*_2_(*ω*), and (c) reflectivity *R*(*ω*) of BaSnS_2_O, SrSnS_2_O, and CaSnS_2_O over 0–35 eV.

The imaginary component, *σ*_2_(*ω*), shown in Fig. 8(b), exhibits pronounced negative minima of approximately −4 fs^−1^ near 4–5 eV, followed by zero crossings around 6–7 eV. At higher photon energies, BaSnS_2_O displays a prominent positive maximum of approximately 3.8 fs^−1^ near 19–20 eV, whereas SrSnS_2_O and CaSnS_2_O exhibit corresponding features of approximately 2.9 and 3.1 fs^−1^ near 24–25 and 28–29 eV, respectively. CaSnS_2_O also shows a brief negative excursion around 25–26 eV. These variations reflect the composition-dependent dispersive characteristics of the optical current response. The sign changes in *σ*_2_(*ω*) describe the reactive optical response rather than variations in direct-current conductivity.

As illustrated in Fig. 8(c), the three compounds exhibit comparable low-energy reflectivities of approximately 0.18, increasing to initial maxima of about 0.37–0.38 within the 6–8 eV region. At higher energies, BaSnS_2_O exhibits the strongest reflectivity maximum of approximately 0.44 near 20–21 eV, whereas SrSnS_2_O and CaSnS_2_O display weaker secondary maxima of approximately 0.18 near 25 eV and 0.16 near 29 eV, respectively. These higher-energy features shift progressively toward larger photon energies across the Ba → Sr → Ca series, accompanied by decreasing peak intensities. Beyond these regions, the reflectivity gradually approaches negligible values. Overall, the spectra demonstrate composition-dependent redistribution of the optical conductivity and reflectivity, particularly at higher photon energies.

#### 3.3.5. Energy-loss function

The calculated polycrystalline-averaged energy-loss functions, *L*(*ω*), of BaSnS_2_O, SrSnS_2_O, and CaSnS_2_O are presented in Fig. 9. The energy-loss function characterizes the energy dissipation experienced by energetic electrons interacting with a material and provides information about its electronic excitation response.^51^ All three compounds exhibit relatively weak energy-loss intensities at low photon energies, followed by pronounced composition-dependent features at higher energies.

**Fig. 9:**
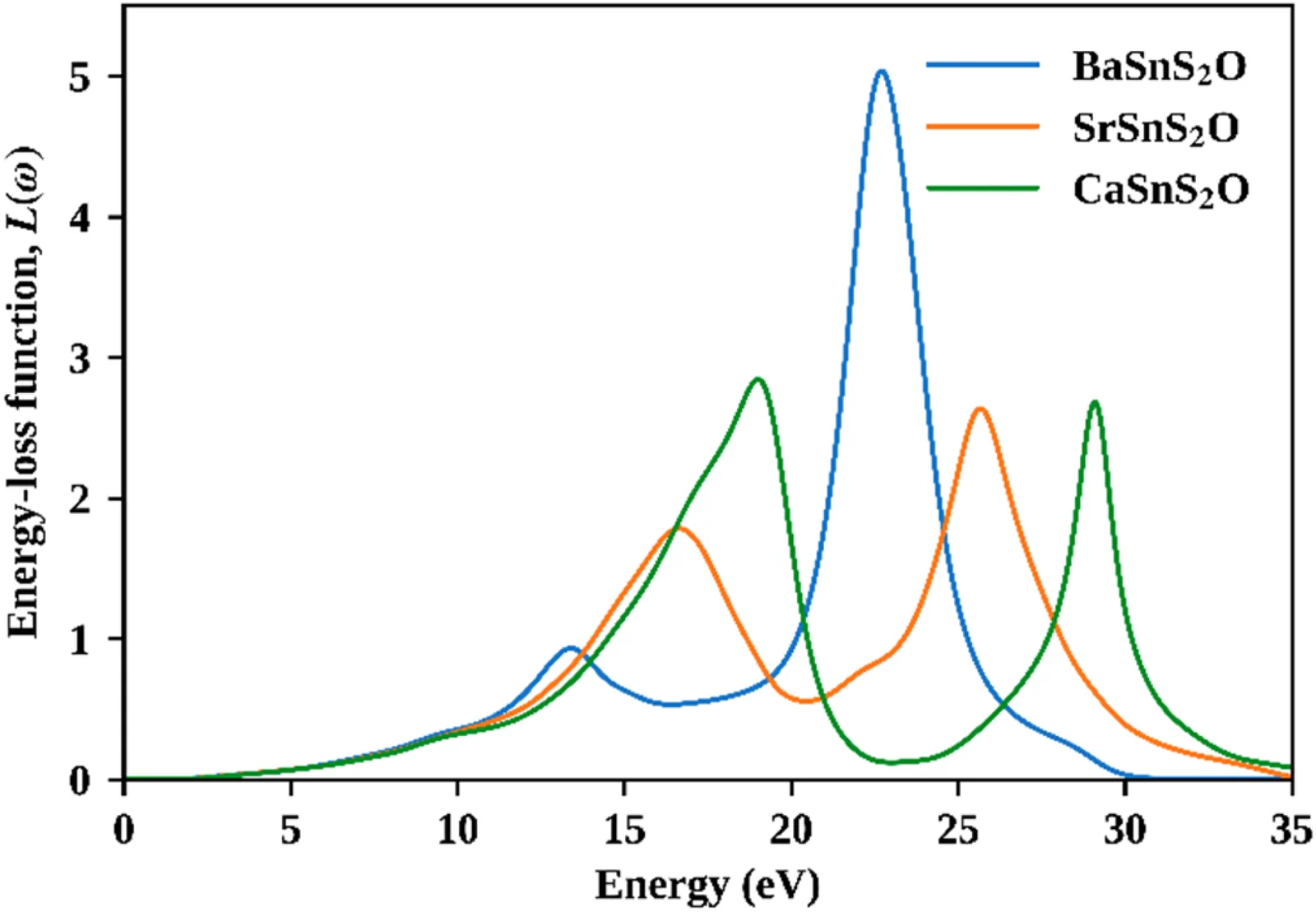
Polycrystalline-averaged energy-loss functions, *L*(*ω*), of BaSnS_2_O, SrSnS_2_O, and CaSnS_2_O over 0–35 eV.

BaSnS_2_O exhibits a weak feature near 13–14 eV and develops the most intense loss peak among the investigated compounds at approximately 22–23 eV, reaching *L*(*ω*) ≈ 5.0. In contrast, SrSnS_2_O displays a broad feature of approximately 1.8 near 16–17 eV, followed by a stronger maximum of about 2.6 near 25–26 eV. CaSnS_2_O exhibits two pronounced peaks, with a maximum of approximately 2.8 near 19 eV and a second peak of about 2.7 near 29 eV. The higher-energy loss features therefore shift progressively toward larger photon energies along the Ba → Sr → Ca series, although their relative intensities differ significantly. Beyond these features, the energy-loss intensities decrease toward the upper end of the investigated energy range.

The variations in peak positions and intensities indicate a composition-dependent redistribution of the electronic energy-loss response. Although pronounced loss maxima may be associated with collective electronic excitations, their identification as bulk-plasmon resonances requires additional dielectric evidence, particularly a near-zero real dielectric response accompanied by sufficiently weak damping. Therefore, the observed maxima are interpreted primarily as characteristic energy-loss features rather than being assigned unambiguously to plasmon resonances.

### 3.4. Elastic and mechanical properties

The elastic and mechanical response of ASnS_2_O (A = Ba, Sr, and Ca) was analyzed using the calculated elastic stiffness constants and the corresponding polycrystalline parameters summarized in Tables 4 and 5. Because the optimized structures exhibit triclinic symmetry, the complete set of 21 independent C_*ij*_ components was considered.

**Table 4:** Calculated independent elastic stiffness constants C_*ij*_ (GPa) of ASnS_2_O (A = Ba, Sr, and Ca)

C_*ij*_ (GPa)	BaSnS_2_O	SrSnS_2_O	CaSnS_2_O
C_11_	85.778	107.747	115.507
C_22_	98.948	69.909	86.261
C_33_	73.226	68.853	74.633
C_44_	25.807	28.267	25.305
C_55_	28.040	27.464	26.545
C_66_	30.321	34.242	38.706
C_12_	30.915	31.104	23.907
C_13_	19.805	20.382	19.268
C_14_	4.526	3.677	5.487
C_15_	0.079	2.907	0.918
C_16_	2.305	−0.171	−0.351
C_23_	41.095	40.433	34.510
C_24_	0.330	2.030	4.893
C_25_	−1.855	0.225	−1.855
C_26_	3.473	2.055	6.635
C_34_	1.789	−2.177	2.337
C_35_	2.854	0.625	−1.420
C_36_	−1.215	−1.728	−0.157
C_45_	−1.999	−1.300	0.948
C_46_	0.653	−0.122	1.526
C_56_	2.735	2.687	3.213

**Table 5:** Calculated polycrystalline elastic and mechanical properties of ASnS_2_O (A = Ba, Sr, and Ca) within the Voigt–Reuss–Hill approximation

Compound	*B*_H_ (GPa)	*G*_H_ (GPa)	*E*_H_ (GPa)	*ν*_H_	(*B*/*G*)	*H*_V_ (GPa)	*A*_U_	*Θ*_D_ (K)
BaSnS_2_O	47.929	27.283	68.795	0.2608	1.757	5.037	0.284	273.5
SrSnS_2_O	47.413	26.520	67.057	0.2643	1.788	4.839	0.737	287.3
CaSnS_2_O	47.225	30.121	74.519	0.2370	1.568	6.148	0.461	330.6

For such low-symmetry systems, elastic mechanical stability requires the full stiffness matrix C to be positive definite, such that
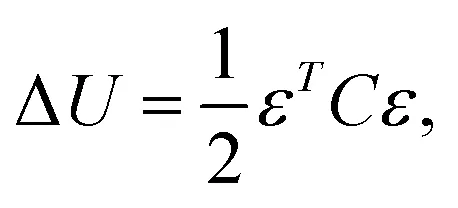


Equivalently, all eigenvalues of *C* must be positive[41]. Diagonalization of the stiffness matrices constructed from Table 4 gives minimum eigenvalues of 22.995, 25.063, and 24.249 GPa for BaSnS_2_O, SrSnS_2_O, and CaSnS_2_O, respectively. Their positive values demonstrate the positive-definite character of the stiffness tensors and hence support the elastic mechanical stability of all three calculated structures.

The individual C_*ij*_ components reveal appreciable directional dependence. In particular, C_11_ increases from 85.778 GPa for BaSnS_2_O to 107.747 GPa for SrSnS_2_O and 115.507 GPa for CaSnS_2_O. In contrast, C_22_ and C_33_ vary non-monotonically, with SrSnS_2_O exhibiting comparatively low values of 69.909 and 68.853 GPa, respectively. This contrasting evolution indicates that A-site substitution does not produce uniform lattice stiffening, but instead redistributes the resistance to deformation among different crystallographic directions. The principal shear constants are generally smaller than the dominant longitudinal constants, indicating that shear distortion is a comparatively softer deformation mode. The finite normal-shear coupling coefficients further reflect the low-symmetry elastic response of these structures.

The effective polycrystalline properties were obtained within the Voigt–Reuss–Hill (VRH) framework,^42^ where
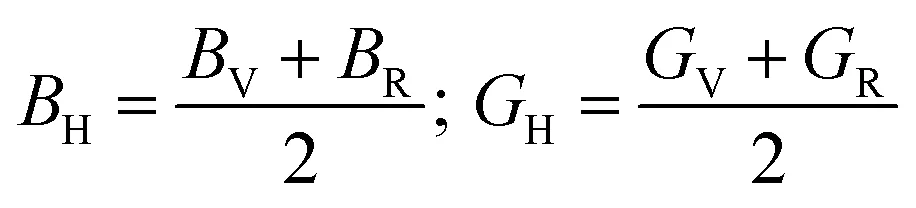

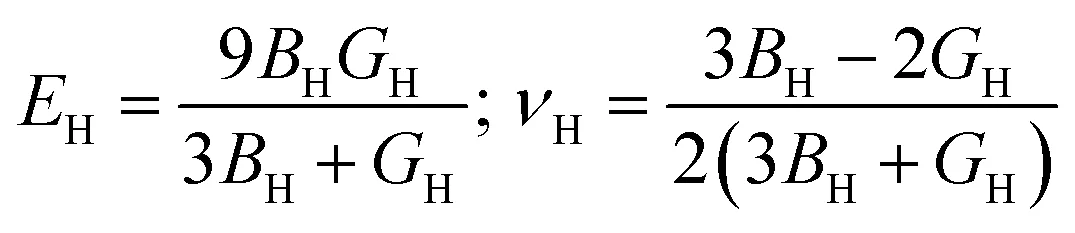


As shown in Table 5, the calculated *B*_H_ values are 47.929, 47.413, and 47.225 GPa for BaSnS_2_O, SrSnS_2_O, and CaSnS_2_O, respectively. Their very small variation shows that the average resistance to hydrostatic compression is nearly insensitive to A-site substitution. In contrast, *G*_H_ varies from 27.283 GPa for BaSnS_2_O and 26.520 GPa for SrSnS_2_O to 30.121 GPa for CaSnS_2_O. CaSnS_2_O also possesses the highest Young's modulus of 74.519 GPa, compared with 68.795 and 67.057 GPa for the Ba and Sr compounds, respectively. Therefore, the substitution effect is more pronounced for shape-changing or distortional deformation than for uniform volume compression, with CaSnS_2_O exhibiting the greatest overall elastic rigidity.

The non-monotonic shear response is particularly evident from the Voigt–Reuss comparison. The calculated *G*_V_/*G*_R_ ratios are 1.047, 1.144, and 1.086 for BaSnS_2_O, SrSnS_2_O, and CaSnS_2_O, respectively, while the corresponding Reuss shear moduli are 26.656, 24.739, and 28.883 GPa. Thus, SrSnS_2_O exhibits both the largest Voigt–Reuss shear separation and the lowest *G*_R_, indicating enhanced compliance and stronger elastic anisotropy. This behavior is consistent with its largest universal anisotropy index (*A*_U_ = 0.737) and with the comparatively low C_22_ and C_33_ values. The Sr anomaly is therefore associated primarily with enhanced elastic anisotropy and directional compliance rather than with a uniform reduction of the overall stiffness. Structurally, this behavior is consistent with the strongly distorted low-symmetry Sn–S/O coordination environment, in which A-site substitution is accompanied by anisotropic changes in local bond lengths and bond angles rather than uniform deformation of the framework. Such local geometric redistribution can contribute to the enhanced directional elastic response observed for SrSnS_2_O.

The relative ductile–brittle tendency was assessed using the Pugh ratio (B/G), with an empirical transition threshold of approximately 1.75.^43^ The calculated B/G values are 1.757 for BaSnS_2_O, 1.788 for SrSnS_2_O, and 1.568 for CaSnS_2_O, indicating a near-boundary response for BaSnS_2_O, the strongest ductile tendency for SrSnS_2_O, and a more brittle-like response for CaSnS_2_O. The corresponding Poisson ratios of 0.2608, 0.2643, and 0.2370, respectively, follow the same relative behavior. Physically, the lower B/G of CaSnS_2_O arises from its comparatively high resistance to shear deformation relative to volume compression.

The theoretical Vickers hardness was evaluated using the Tian relation,^44^
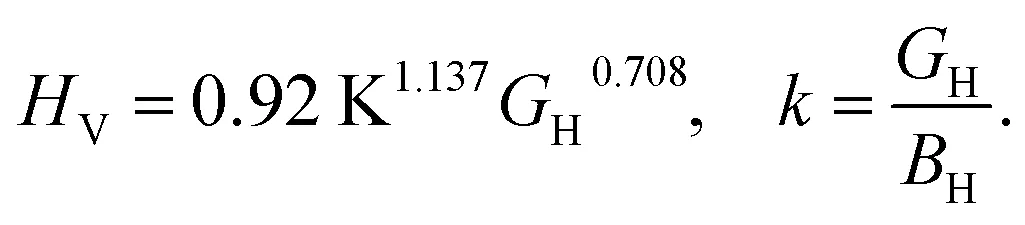


The resulting hardness values are 5.037, 4.839, and 6.148 GPa for BaSnS_2_O, SrSnS_2_O, and CaSnS_2_O, respectively. The highest *H*_v_ of CaSnS_2_O is consistent with its largest *G*_H_ and *E*_H_, since resistance to shear deformation plays a central role in the elastic-modulus-based hardness description.

Elastic anisotropy was quantified using the universal anisotropy index
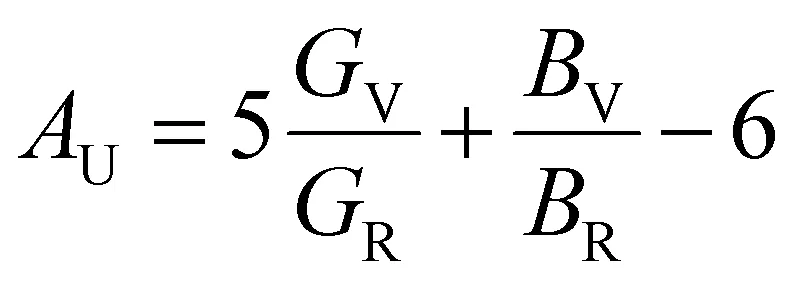
where *A*_U_ = 0 represents an elastically isotropic medium.^45^ The calculated values of 0.284, 0.737, and 0.461 for BaSnS_2_O, SrSnS_2_O, and CaSnS_2_O confirm finite elastic anisotropy in all three systems. SrSnS_2_O exhibits the largest anisotropy, consistent with its enhanced directional compliance and the larger difference between its Voigt and Reuss shear responses.

Finally, the elastic Debye temperature increases monotonically from 273.5 K for BaSnS_2_O to 287.3 K for SrSnS_2_O and 330.6 K for CaSnS_2_O. Within the elastic Debye framework, *Θ*_D_ is related to the mean acoustic velocity and therefore reflects the combined influence of elastic rigidity and mass density.^46^ The higher *Θ*_D_ of CaSnS_2_O is thus consistent with its comparatively larger shear and Young's moduli and indicates a higher characteristic acoustic-vibrational energy scale.

The strain-amplitude test (Table S3) shows that several stiffness constants, including C_22_ of SrSnS_2_O, vary appreciably with the maximum strain amplitude. Recomputing the Voigt–Reuss–Hill quantities from the constants obtained at 0.002 and 0.004 (Table S3b of the SI) shows that the highest Hill shear modulus of CaSnS_2_O is retained at all three amplitudes, although its margin changes. In contrast, the lowest Hill shear modulus, the highest Pugh ratio and the highest universal anisotropy index of SrSnS_2_O, the lowest Pugh ratio of CaSnS_2_O, and the near invariance of the Hill bulk modulus are most clearly seen at 0.003. These features should therefore be regarded as indicative rather than converged.

Overall, the calculated elastic and mechanical observables do not exhibit a single systematic Ba → Sr → Ca trend. The Hill bulk modulus remains nearly invariant, whereas the Debye temperature increases monotonically across the series. In contrast, the shear modulus, Young's modulus, Pugh ratio, theoretical hardness, and universal anisotropy index vary non-monotonically, with SrSnS_2_O showing a distinct intermediate-composition response. At the reference strain amplitude of 0.003, CaSnS_2_O exhibits the highest calculated elastic rigidity and theoretical hardness, whereas SrSnS_2_O shows the highest Pugh ratio and universal anisotropy index. However, the latter rankings are sensitive to the applied strain amplitude.

### 3.5. Energetic and dynamical stability assessment

#### 3.5.1. Formation energies and energetic comparison with selected competing phases

The energetic tendency of the optimized ASnS_2_O (A = Ba, Sr, and Ca) structures was first evaluated relative to the corresponding elemental reference states. The formation energy per formula unit was calculated as
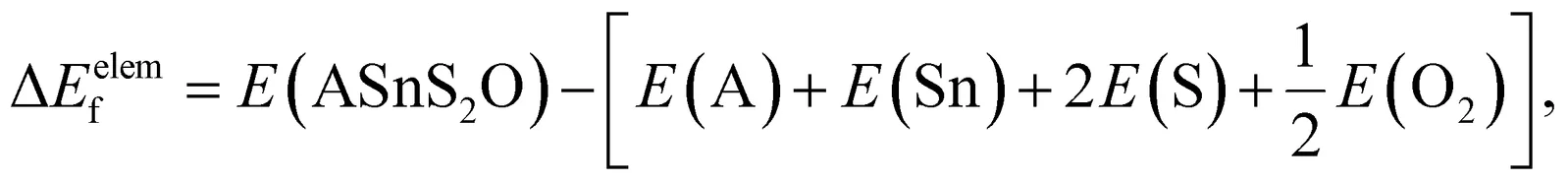
where *E*(ASnS_2_O) is the total energy of the fully optimized oxysulfide per formula unit, and *E*(A), *E*(Sn), *E*(S), and *E*(O_2_) are the corresponding elemental reference energies. Each optimized ASnS_2_O simulation cell contains 20 atoms, corresponding to four formula units; therefore, the calculated cell energies were normalized by four before evaluating the formation energies.

The calculated elemental-reference formation energies are −8.235, −8.383, and −8.236 eV per f.u. for BaSnS_2_O, SrSnS_2_O, and CaSnS_2_O, respectively, corresponding to −1.647, −1.677, and −1.647 eV per atom. These negative values indicate that formation of the investigated ASnS_2_O structures is energetically favorable relative to the selected elemental reference states within the present DFT framework. Among the three compositions, SrSnS_2_O exhibits the most negative elemental-reference formation energy. However, a negative formation energy relative to the elements alone does not establish stability against decomposition into alternative binary or ternary phases. Therefore, additional comparisons were performed using selected competing-phase assemblages.

Stoichiometrically balanced competing-phase assemblages were constructed to assess the energetic stability of ASnS_2_O against selected decomposition channels:AO + SnS_2_ → ASnS_2_O,
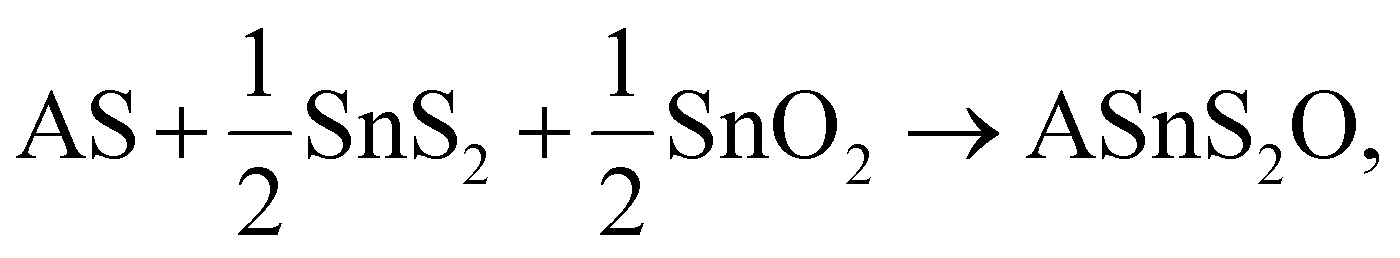
and
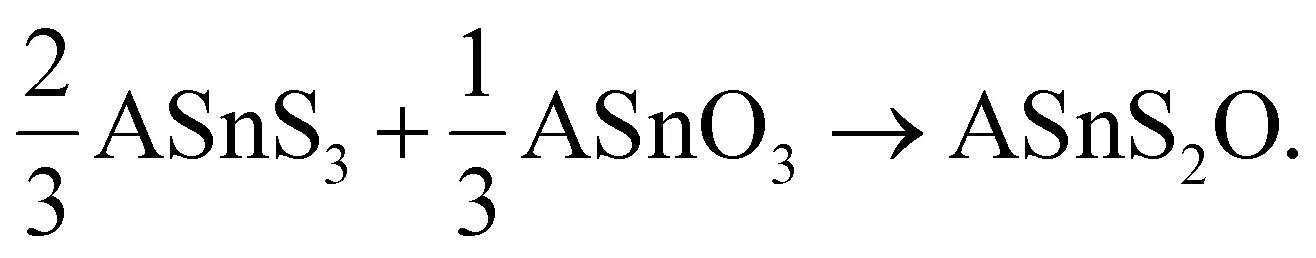


Because the calculated cells of the target and competing phases contain different numbers of formula units, all total energies were normalized to an eV-per-formula-unit basis before comparison. The relative energy with respect to each competing assemblage was calculated as
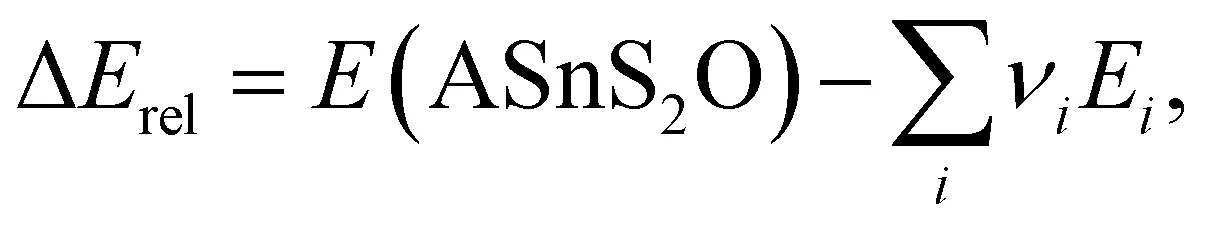
where *E*_*i*_ is the normalized total energy of competing phase *i*, and *ν*_*i*_ is its stoichiometric coefficient. Under this convention, a negative Δ*E*_rel_ indicates that the investigated ASnS_2_O configuration is lower in energy than the specified competing assemblage, whereas a positive value indicates that the competing assemblage is lower in energy.

As shown in Table 6, all three ASnS_2_O compositions exhibit negative relative energies with respect to the AO + SnS_2_ assemblage, with values of −1.342, −0.830, and −0.298 eV per f.u. for BaSnS_2_O, SrSnS_2_O, and CaSnS_2_O, respectively. Thus, the investigated ASnS_2_O configurations are lower in energy than the corresponding AO + SnS_2_ combinations. The magnitude of this energetic preference decreases from BaSnS_2_O to CaSnS_2_O.

**Table 6:** Calculated elemental-reference formation energies and relative energies of ASnS_2_O with respect to selected competing-phase assemblages^a^

Compound	E_ASnS_2_O_ (eV per f.u.)	Δ*E*^elem^_f_ (eV per f.u.)	Δ*E*^elem^_f_ (eV per atom)	AO + SnS_2_ (eV per f.u.)	As + ½SnS_2_ + ½SnO_2_ (eV per f.u.)	⅔ASnS_3_ + ⅓ASnO_3_ (eV per f.u.)
BaSnS_2_O	−1781.9979	−8.235	−1.647	−1.342	+0.978	+0.488
SrSnS_2_O	−1925.8906	−8.383	−1.677	−0.830	+1.114	+0.519
CaSnS_2_O	−2084.8769	−8.236	−1.647	−0.298	+1.234	+0.608

a *E*_ASnS_2_O_ denotes the CASTEP total energy normalized per formula unit. Negative Δ*E*_rel_ values indicate that the investigated ASnS_2_O configuration is lower in energy than the specified competing assemblage, whereas positive values indicate the opposite.

In contrast, positive relative energies are obtained for the AS + ½SnS_2_ + ½SnO_2_ assemblage, with values of +0.978, +1.114, and +1.234 eV per f.u. for BaSnS_2_O, SrSnS_2_O, and CaSnS_2_O, respectively. These values indicate that this selected competing-phase combination is lower in energy than the investigated ASnS_2_O configurations.

Similarly, comparison with the ⅔ASnS_3_ + ⅓ASnO_3_ assemblage gives positive relative energies of +0.488, +0.519, and +0.608 eV per f.u. for BaSnS_2_O, SrSnS_2_O, and CaSnS_2_O, respectively. Therefore, the investigated ASnS_2_O configurations also lie above this particular ternary competing-phase combination.

The negative elemental-reference formation energies indicate energetic favorability relative to the selected elemental states. However, the investigated ASnS_2_O configurations lie above the AS + ½SnS_2_ + ½SnO_2_ and ⅔ASnS_3_ + ⅓ASnO_3_ assemblages in energy, indicating that decomposition into these combinations is energetically favorable within the present DFT treatment. Their lower energies relative to AO + SnS_2_ therefore do not establish stability against decomposition into other phases. Whether these configurations can persist as metastable phases remains unresolved because decomposition barriers and kinetic lifetimes were not evaluated.

The present analysis is restricted to selected competing phases and does not constitute a complete convex-hull calculation. Although the identified lower-energy assemblages indicate that the investigated configurations are not equilibrium ground states within the present energetic comparison, determining the equilibrium decomposition products and exact energies above the convex hull requires consideration of all relevant competing phases. The reported relative energies should therefore be interpreted as differences with respect to specified assemblages rather than as complete convex-hull distances.

#### 3.5.2. *Γ*-Point vibrational analysis

The *Γ*-point vibrational frequencies of the fully optimized ASnS_2_O (A = Ba, Sr, and Ca) structures are presented in Fig. 10. For each 20-atom cell, 60 vibrational modes are obtained at the Brillouin-zone center. No imaginary (negative) frequencies are observed for BaSnS_2_O, SrSnS_2_O, or CaSnS_2_O. The three lowest-frequency modes occur at or very close to zero frequency, consistent with the translational acoustic modes expected at *Γ*.

**Fig. 10:**
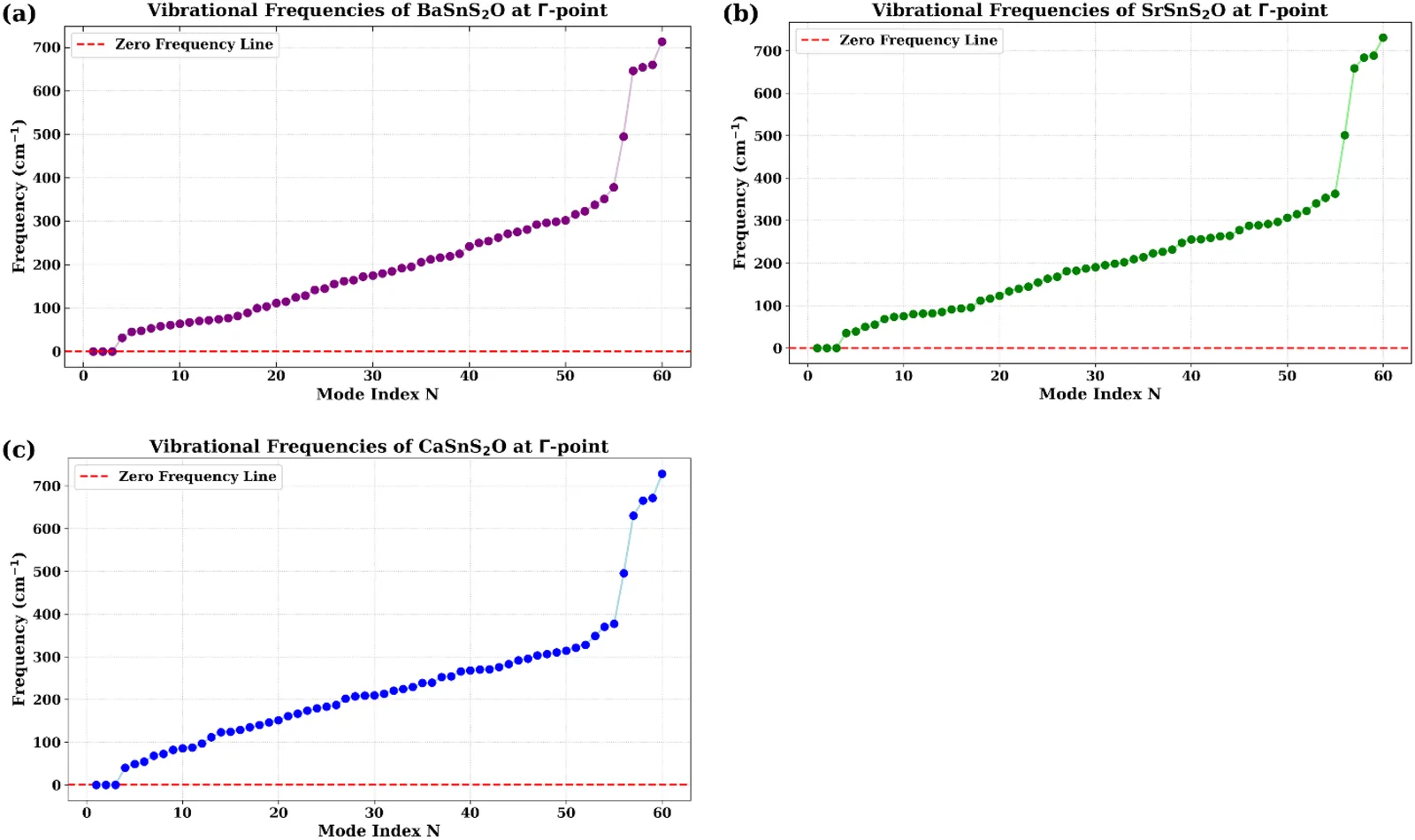
*Γ*-Point vibrational frequencies of the fully optimized (a) BaSnS_2_O, (b) SrSnS_2_O, and (c) CaSnS_2_O structures. The dashed red horizontal line denotes zero frequency; connecting lines are included only as guides to the eye.

The remaining modes exhibit positive frequencies throughout the calculated *Γ*-point spectrum. Although modest composition-dependent shifts are observed among individual modes, the overall vibrational-frequency distributions remain broadly similar across the Ba–Sr–Ca series, and no zone-center soft mode is evident in any of the optimized structures.

The absence of imaginary *Γ*-point frequencies provides evidence of local dynamical stability with respect to zone-center lattice distortions. However, because the present vibrational analysis is restricted to the *Γ* point, possible instabilities at finite wave vectors cannot be excluded. Accordingly, the present results support local dynamical stability at the Brillouin-zone center rather than complete dynamical stability throughout the Brillouin zone. A full phonon-dispersion calculation would be required for a comprehensive assessment of dynamical stability.

### 3.6. Band-edge alignment for water-redox screening

The band-edge alignment of ASnS_2_O (A = Ba, Sr, and Ca) relative to the water-redox potentials was examined as an initial thermodynamic screening. The band-edge positions were estimated using the Mulliken electronegativity approach, which relates the absolute electronegativity of a semiconductor to its band-gap energy.^48,52^ For ASnS_2_O, the compound electronegativity was obtained as*X* = (*X*_A_*X*_Sn_*X*_S_^2^*X*_O_)^1/5^and the band-edge potentials were estimated from
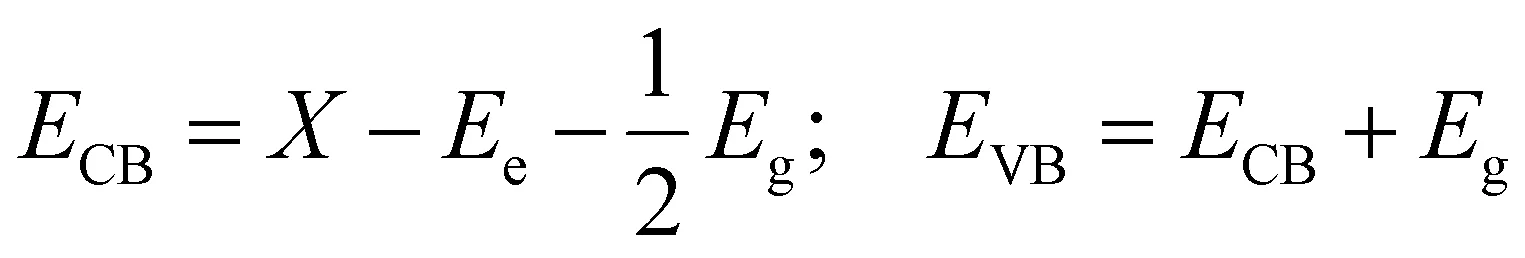
where *E*_e_ = 4.50 eV represents the free-electron energy on the NHE scale. This electronegativity-based treatment provides an approximate means of estimating semiconductor band-edge positions when direct electrochemical measurements are unavailable.

The present PBE–Mulliken band-edge positions should be regarded as approximate screening estimates rather than quantitatively precise electrochemical potentials. GGA-PBE can underestimate semiconductor band gaps, and the resulting uncertainty propagates directly into the estimated CBM and VBM positions. In addition, the Mulliken electronegativity approach introduces further uncertainty, while the possible influence of alternative S/O configurations is not included in the present estimates. Surface termination, solvent effects, interfacial band bending, and catalytic overpotentials are also beyond the present model. Accordingly, the numerical offsets from the water-redox levels are interpreted qualitatively rather than as precise energetic margins. To illustrate the sensitivity of the estimated band-edge positions, a hypothetical ±0.2 eV variation in the compound electronegativity (*χ*), at a fixed band gap, would shift both the CBM and VBM potentials by ±0.2 V. This illustrative variation highlights the uncertainty associated with the absolute band-edge positions, particularly for BaSnS_2_O, whose nominal VBM lies close to the oxygen evolution reaction (OER) threshold.

At pH = 0, the thermodynamic potentials for H^+^/H_2_ and O_2_/H_2_O are 0 and +1.23 V *versus* NHE, respectively. For qualitative screening of the hydrogen evolution reaction (HER), the CBM should lie on the negative-potential side of the H^+^/H_2_ level, whereas the OER requires the VBM to lie on the positive-potential side of the O_2_/H_2_O level.^53^ These conditions provide an initial thermodynamic criterion for evaluating the nominal band-edge alignment of the materials.

The estimated CBM values of BaSnS_2_O, SrSnS_2_O, and CaSnS_2_O are −0.175, −0.237, and −0.269 V *versus* NHE, respectively (Table 7). Within the common PBE–Mulliken screening framework, all three nominal CBM positions lie on the negative-potential side of the H^+^/H_2_ level and shift progressively toward more negative potentials along the Ba → Sr → Ca sequence. Because the absolute offsets are subject to the methodological uncertainties discussed above, this evolution is interpreted only as a qualitative trend in the reduction-side band-edge alignment (Fig. 11).

**Table 7:** Nominal PBE–Mulliken band-edge estimates of ASnS_2_O (A = Ba, Sr, and Ca) at pH = 0

Material	*χ* (eV)	*E*_g_ (PBE)(eV)	*E*_CB_ (V *vs.* NHE)	*E*_VB_ (V *vs.* NHE)
BaSnS_2_O	5.055	1.461	−0.175	1.286
SrSnS_2_O	5.127	1.727	−0.237	1.490
CaSnS_2_O	5.195	1.928	−0.269	1.659

**Fig. 11:**
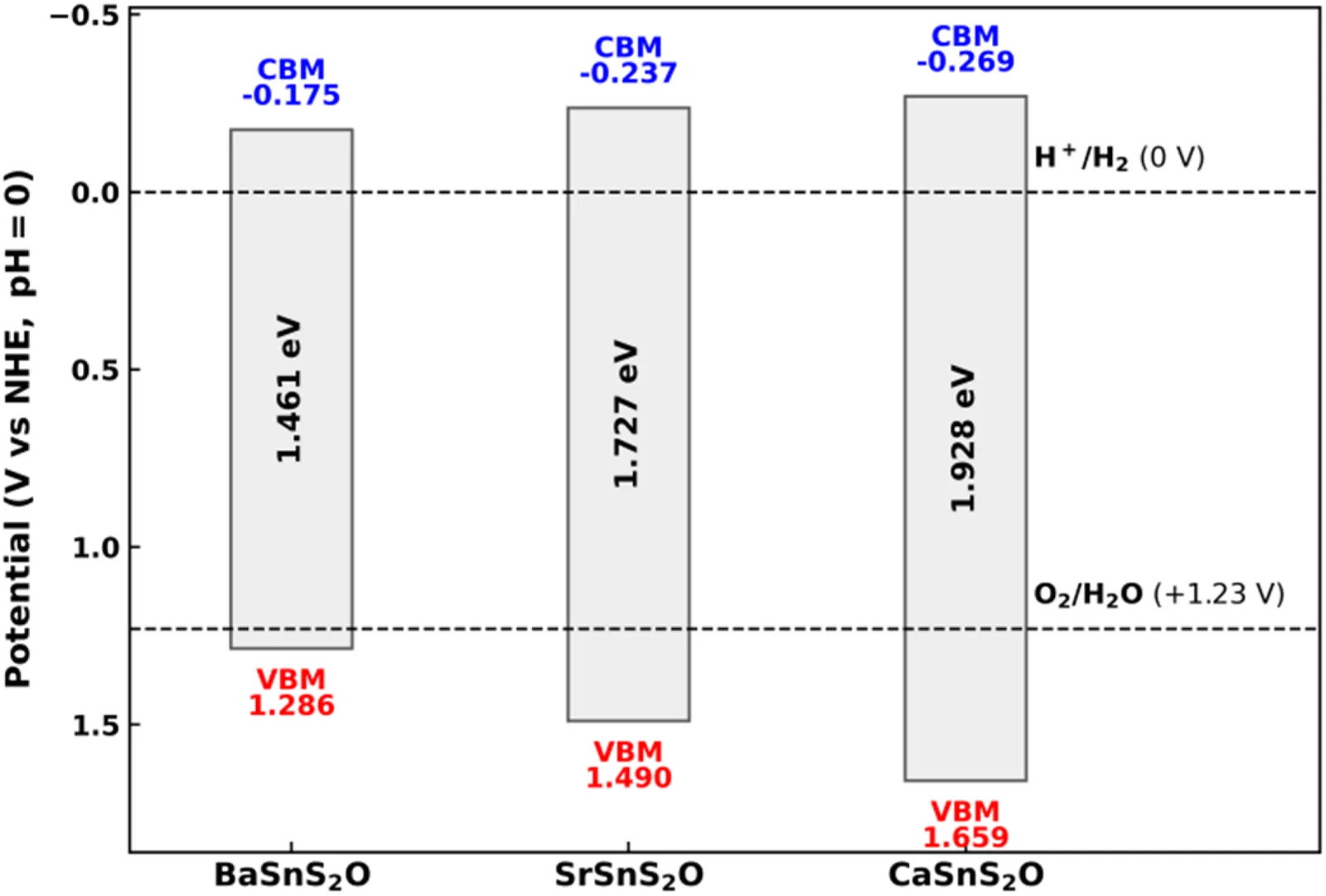
Nominal PBE–Mulliken band-edge alignment of ASnS_2_O (A = Ba, Sr, and Ca) relative to the H^+^/H_2_ and O_2_/H_2_O redox levels at pH = 0.

The estimated VBM positions also shift toward more positive potentials along the Ba → Sr → Ca sequence, increasing from 1.286 V for BaSnS_2_O to 1.490 V for SrSnS_2_O and 1.659 V for CaSnS_2_O. Within the present screening framework, these nominal positions lie on the positive-potential side of the O_2_/H_2_O level. However, the corresponding offsets should not be interpreted as quantitatively established oxidation-side energetic margins. In particular, BaSnS_2_O lies close to the oxidation threshold relative to the expected uncertainty of the present approach and should therefore be regarded as borderline within this screening framework.

The simultaneous nominal shift of the CBM toward more negative potentials and the VBM toward more positive potentials from BaSnS_2_O to CaSnS_2_O arises from the combined variation of the compound electronegativity and the calculated PBE band gap rather than from a rigid displacement of both band edges. Within the present screening framework, the relative band-edge alignment follows the qualitative trend CaSnS_2_O > SrSnS_2_O > BaSnS_2_O. This ordering is used only as a relative screening trend and not as a quantitative comparison of HER or OER driving forces.

To further evaluate the sensitivity of the water-redox screening results to the band-gap treatment, the Sn-5p PBE+*U* band gaps obtained with *U* = 2–4 eV were incorporated into the same Mulliken electronegativity-based model (Table S8, SI). The compound electronegativities derived from literature-based ionization-energy and electron-affinity data were retained consistently with the main-text calculations. Across the investigated *U* range, all three compounds maintain nominal CBM positions on the negative-potential side of the H^+^/H_2_ level and VBM positions on the positive-potential side of the O_2_/H_2_O level, preserving the qualitative alignment trend CaSnS_2_O > SrSnS_2_O > BaSnS_2_O. Since the band-edge estimates were obtained using the same electronegativity-based expressions with modified band gaps, the resulting shifts represent model-dependent sensitivity rather than independently calculated absolute PBE+*U* band-edge positions. In particular, BaSnS_2_O remains close to the oxidation-side threshold, where methodological uncertainties may affect the inferred alignment. Accordingly, these findings support qualitative water-redox screening rather than quantitatively reliable HER/OER driving forces or confirmed practical water-splitting feasibility.

It should further be emphasized that nominally suitable band-edge positions constitute only an initial thermodynamic screening criterion and do not demonstrate photocatalytic activity. Actual water-splitting performance additionally depends on photogenerated-carrier separation and recombination, surface reaction kinetics, catalytic overpotentials, active surface sites, chemical stability, solvent effects, and semiconductor–electrolyte interfacial phenomena. Consequently, the present PBE–Mulliken analysis and accompanying PBE+*U* sensitivity assessment are limited to qualitative band-edge screening. Higher-level electronic-structure calculations together with explicit surface and electrochemical modeling would be required for a quantitatively reliable assessment of water-splitting energetics.

### 3.7. Material characteristics and applications

The calculated results reveal a composition-dependent structure–property evolution that is monotonic for some observables but non-monotonic for others across the ASnS_2_O (A = Ba, Sr, and Ca) mixed-anion series. For the same ordered S → O substitution motif, structural relaxation yields low-symmetry triclinic configurations, with the unit-cell volume decreasing from 446.001 Å^3^ for BaSnS_2_O to 418.804 Å^3^ for SrSnS_2_O and 394.341 Å^3^ for CaSnS_2_O. This contraction is accompanied by anisotropic lattice deformation and redistribution of the local Sn–S/O coordination. Consistent with these structural modifications, the indirect band gap increases from 1.461 eV for BaSnS_2_O to 1.727 eV for SrSnS_2_O and 1.928 eV for CaSnS_2_O. The PDOS further shows that the upper valence states are dominated mainly by S/O-p orbitals, whereas the conduction-band edge is largely Sn-derived, indicating that the electronic response is governed primarily by the Sn–anion framework.

The optical and mechanical responses provide complementary evidence of composition-dependent tuning. The polycrystalline-averaged optical spectra reveal pronounced composition-dependent redistribution of dielectric, absorption, optical conductivity, reflectivity, and energy-loss features, with calculated absorption coefficients reaching the order of 10^5^ cm^−1^. The dominant high-energy absorption maxima lie beyond the visible and near-ultraviolet regions and therefore should not be interpreted as direct evidence of efficient solar-spectrum absorption. Mechanically, the positive-definite triclinic stiffness matrices support elastic mechanical stability within the present calculations.^41^ The mechanical observables do not follow a single monotonic Ba → Sr → Ca progression. The Hill bulk modulus remains nearly invariant, whereas the Debye temperature increases monotonically across the series. In contrast, the shear modulus, Young's modulus, Pugh ratio, theoretical hardness, and universal elastic anisotropy index vary non-monotonically. At the reference strain amplitude of 0.003, CaSnS_2_O exhibits the largest shear and Young's moduli, theoretical hardness, and Debye temperature, whereas SrSnS_2_O shows the highest Pugh ratio and universal elastic anisotropy index. The distinct SrSnS_2_O response is consistent with its enhanced elastic anisotropy and directional compliance, as evidenced by the Voigt–Reuss shear-modulus separation and universal anisotropy index. These results demonstrate that A-site variation modifies shear resistance and directional rigidity more strongly than the nearly unchanged resistance to hydrostatic compression. Several of these mechanical trends depend on the strain amplitude and are therefore indicative (Section 3.4 and Table S3b).

From an application perspective, the calculated PBE–Mulliken band-edge alignment provides an initial thermodynamic basis for further assessment of ASnS_2_O in photocatalytic and photoelectrochemical water-redox processes. Within this screening framework,^48^ the nominal band-edge alignment follows the relative trend CaSnS_2_O > SrSnS_2_O > BaSnS_2_O, while BaSnS_2_O lies closest to the oxidation-side threshold and should be regarded as borderline within the methodological uncertainty. These results are interpreted qualitatively rather than as quantitative HER or OER driving-force margins. Within the investigated *U* = 2–4 eV range, the Sn-5p PBE+*U* calculations preserve the relative band-gap ordering, while the corresponding Mulliken-based screening retains the nominal water-redox alignment trend (Tables S7 and S8, SI). However, the weak lowest-conduction-band dispersion and the large, strongly direction-dependent carrier effective masses indicate that favorable band-edge alignment alone does not establish efficient carrier transport or photocatalytic performance. Overall, the ASnS_2_O series demonstrates how mixed-anion coordination together with A-site variation can tune structural, electronic, optical, mechanical, and redox characteristics within a single semiconductor family. The present results therefore support further water-redox and carrier-transport assessment of the ASnS_2_O series rather than demonstrating its suitability for practical water splitting, while broader optoelectronic or photovoltaic utilization requires dedicated assessment.

## 4. Conclusion

In this work, the structural, electronic, optical, elastic, and nominal water-redox characteristics of mixed-anion ASnS_2_O (A = Ba, Sr, Ca) were examined by density functional theory within GGA–PBE using a common ordered S/O motif. The relaxed primary configurations adopt triclinic P1 symmetry, with an 11.6% unit-cell contraction from BaSnS_2_O (446.001 Å^3^) to CaSnS_2_O (394.341 Å^3^). Their indirect band gaps increase from 1.461 to 1.727 and 1.928 eV, respectively. However, selected alternative S/O configurations exhibit substantial variations in both energy and band gap, and the primary configurations cannot be regarded as established global ground states. Fixed-volume calculations and Mulliken overlap analysis indicate that volume contraction alone cannot explain the band-gap widening; changes in A-site chemistry and local Sn–S/O coordination also contribute. Sn-5p PBE+*U* calculations retain the relative gap ordering but do not validate the absolute gap values.

Polycrystalline-averaged optical spectra show peak absorption coefficients of 3.04 × 10^5^, 2.35 × 10^5^, and 2.56 × 10^5^ cm^−1^ at 18.8, 23.6, and 27.8 eV, respectively, well beyond the visible region. Positive-definite elastic stiffness matrices support elastic mechanical stability within the calculated structures, although several derived mechanical trends are strain-amplitude sensitive. CaSnS_2_O has the highest theoretical hardness (6.148 GPa), whereas SrSnS_2_O shows the largest elastic anisotropy in the reference strain dataset.

At pH = 0, nominal PBE–Mulliken band-edge estimates suggest progressively more favorable qualitative water-redox alignment from Ba to Ca, with BaSnS_2_O closest to the oxidation threshold. Nevertheless, the investigated configurations lie above selected competing decomposition assemblages, and the absence of imaginary *Γ*-point phonon frequencies does not establish full dynamical stability. Thus, these findings demonstrate composition-dependent structure–property tuning in ordered ASnS_2_O while emphasizing the need for broader phase-stability searches, accurate absolute band edges, carrier-transport assessments, and explicit surface-reaction studies before practical photocatalytic feasibility can be established.

## Author contributions

Golam Morshed: conceptualization, investigation, simulation and analysis, visualization, writing – original draft, and writing – review and editing, formal analysis; S. M. Anowarul Haque: supervision, writing – original draft, and writing – review and editing, formal analysis; Abdul Wahed: supervision, writing – original draft and writing – review and editing; formal analysis, Joyonta Das: writing – original draft and writing – review and editing; Md Sabbir Hossain: writing – original draft and writing – review and editing; Mohammad Sefat: visualization, writing – review and editing; Fahmid Hasan Fahim: writing – review and editing, formal analysis; Tawsif Md Samiul Hossain: writing – review and editing, formal analysis.

## Conflicts of interest

The authors declare no competing interests.

## Supplementary Material

RA-OLF-D6RA07784F-s001

RA-OLF-D6RA07784F-s002

## Data Availability

The structural data and CASTEP input files are provided in the supplementary information (SI) and Data. Additional simulation data are available from the corresponding author upon reasonable request. Supplementary information is available. See DOI: https://doi.org/10.1039/d6ra07784f.
