# Supplementary material for: Mixed-anion engineering of ASnS2O (A = Ba, Sr, Ca): first-principles insights into structure–property tuning and water-redox band-edge screening
Source: RSC Adv. 2026 Nov 13. Online ahead of print. doi: 10.1039/d6ra07784f (PMC13760272; doi:10.1039/d6ra07784f)
Supplement: Supplementary file 1 [file RA-OLF-D6RA07784F-s002.pdf]

Supplementary Information (SI) for RSC Advances.
This journal is © The Royal Society of Chemistry

Mixed-Anion Engineering of $ASnS_2O$ (A = Ba, Sr, Ca): First-Principles Insights into Structure–Property Tuning and Water-Redox Band-Edge Screening

Golam Morshed[1*], Anowarul Haque[1], Abdul Wahed[1], Joyonta Das[1], Md Sabbir Hossain[1], Mohammad Sefat[1], Fahmid Hasan Fahim[1], Tawsif Md Samiul Hossain[1]

[1]Department of EEE, Mymensingh Engineering College (University of Dhaka), Mymensingh, Bangladesh

[*]Corresponding author: E-mail: riyamahmed6350@gmail.com

Supplemental Material

I. A-site coordination analysis

II. Quantitative Shannon-radius and unit-cell-volume analysis

III. Strain-amplitude sensitivity of the elastic constants

IV. Convergence with respect to k-point sampling and plane-wave cutoff

V. Optical Properties: Dielectric Tensor and Polarization-Dependent Response

VI. Band-edge dispersion and CASTEP Mulliken populations

VII. Reciprocal fixed-volume and local-geometry analysis

VIII. Sn-5p DFT+U (PBE+U) band-gap and water-redox screening sensitivity

IX. S/O configuration enumeration and screening

X. Comparison of $CaSnS_2O$ Configurations Derived from Different Parent Frameworks

XI. Structural Sources and Computational Details of Reference Phases

I. A-site coordination analysis

The A-site coordination environments of the fully relaxed $BaSnS_2O$, $SrSnS_2O$, and $CaSnS_2O$ structures were evaluated using periodic-boundary-condition (PBC)-corrected A–O/S distance sorting. The same distance-sorting approach employed for the Sn first-shell analysis in the main manuscript was applied independently to each crystallographically inequivalent A site. Periodic images of the O and S atoms were included, and the A–anion distances were sorted in ascending order. For each A site, the first coordination shell was identified from the separation between the near-neighbor group and the subsequent distance shell. Because the relaxed structures belong to the low-symmetry P1 space group, the four A sites are crystallographically inequivalent and exhibit strongly distorted, site-dependent coordination environments.

Table S1. Site-resolved PBC-corrected A-site coordination environments of the fully relaxed $ASnS_2O$ (A = Ba, Sr, and Ca) structures.

Compound	A site	Coordination number (CN)	First-shell distance range (Å)	Next contact (Å)
$BaSnS_2O$	A1	9	3.092–3.622	5.060
$BaSnS_2O$	A2	9	2.690–3.659	4.508
$BaSnS_2O$	A3	9	2.849–3.494	4.873
$BaSnS_2O$	A4	9	2.642–3.679	4.449
$SrSnS_2O$	A1	9	2.519–3.773	4.396
$SrSnS_2O$	A2	10	2.463–3.727	4.466

Compound	A site	Coordination number (CN)	First-shell distance range (Å)	Next contact (Å)
$SrSnS_2O$	A3	9	2.941–3.668	4.822
$SrSnS_2O$	A4	7	2.915–3.582	4.526
$CaSnS_2O$	A1	9	2.431–3.744	4.237
$CaSnS_2O$	A2	10	2.345–3.520	4.313
$CaSnS_2O$	A3	9	2.647–3.892	4.445
$CaSnS_2O$	A4	7	2.753–3.850	4.641

A1–A4 denote the four crystallographically inequivalent A sites in each relaxed P1 structure.

The site-resolved analysis gives average A-site coordination numbers of 9.00, 8.75, and 8.75 for $BaSnS_2O$, $SrSnS_2O$, and $CaSnS_2O$, respectively. All four Ba sites are nine-coordinate, whereas the Sr and Ca structures exhibit heterogeneous seven- to ten-coordinate environments. These results demonstrate that the relaxed P1 structures do not possess a uniform ideal coordination polyhedron; instead, their A-site environments are strongly distorted, with average coordination lying close to nine.

II. Quantitative Shannon-radius and unit-cell-volume analysis

To quantify the A-site size trend, Shannon effective ionic radii [1] were compared with the optimized unit-cell volumes. Because the relaxed A-site environments are distorted and site-dependent, the CN = 8 Shannon radii are used here as a common effective size descriptor rather than as evidence of exact eightfold coordination at every A site. The CN = 6 and CN = 12 Shannon radius sets were also examined for comparison.

Table S2. Shannon effective ionic radii [1] and their relationship with the optimized ASnS₂O unit-cell-volume trend.

Shannon CN	Ba^{2+} radius (Å)	Sr^{2+} radius (Å)	Ca^{2+} radius (Å)	dV/dr, Ba→Sr ($Å^3$ $Å^{-1}$)	dV/dr, Sr→Ca ($Å^3$ $Å^{-1}$)	Relative slope spread (%)
6	1.35	1.18	1.00	160.0	135.9	16.3
8	1.42	1.26	1.12	170.0	174.7	2.8
12	1.61	1.44	1.34	160.0	244.6	41.8

The relative slope spread is defined as ∣m_1−m_2∣/[(m_1+m_2)/2] ×100%, where m_1 and m_2 are the Ba→Sr and Sr→Ca volume–radius slopes, respectively.

The optimized unit-cell volumes are 446.001, 418.804, and 394.341 Å^3 for $BaSnS_2O$, $SrSnS_2O$, and $CaSnS_2O$, respectively. Using the CN = 8 Shannon radii of 1.42, 1.26, and 1.12 Å gives volume–radius slopes of 170.0 Å^3 Å^{-1} for Ba→Sr and 174.7 Å^3 Å^{-1} for Sr→Ca. These slopes differ by only approximately 2.8%, and a linear fit of the optimized volumes against the CN = 8 radii gives R^2=0.99994. By comparison, the CN = 6 and CN = 12 radius sets produce substantially larger relative slope spreads of approximately 16% and 42%, respectively. Thus, among the Shannon radius sets considered, the CN = 8 values provide the most internally consistent common effective size scale for describing the Ba→Sr→Ca volume trend, although the relaxed local A-site environments are not uniformly eight-coordinate.

From $BaSnS_2O$ to $CaSnS_2O$, the CN = 8 Shannon radius decreases from 1.42 to 1.12 Å, corresponding to a reduction of approximately 21.1%. Over the same sequence, the optimized unit-cell volume decreases from 446.001 to 394.341 Å^3, corresponding to a contraction of approximately 11.6%. The equivalent isotropic linear contraction,

$$1 - \left(\frac{V_{Ca}}{V_{Ba}}\right)^{\frac{1}{3}},$$

is approximately 4.0%. The substantially smaller linear lattice contraction compared with the reduction in the A-site ionic radius indicates that the A-site size variation is not accommodated by uniform compression alone. Instead, a substantial fraction of the size change is accommodated through anisotropic lattice deformation and local rearrangement of the Sn– (S, O) framework.

III. Strain-amplitude sensitivity of the elastic constants

To examine the sensitivity of the calculated elastic constants to the applied strain amplitude, the maximum strain amplitude was varied from the reference value of 0.003 to 0.002 and 0.004 while retaining the same four-step elastic-constant calculation procedure. The complete set of 21 independent elastic stiffness constants, C_{ij}, obtained at the three strain amplitudes is summarized in Table S3 for $BaSnS_2O$, $SrSnS_2O$, and $CaSnS_2O$. The comparison reveals that several elastic components exhibit strain-amplitude sensitivity, with the small off-diagonal terms showing the largest relative variations and occasional sign changes, while several diagonal constants (C_{22} of $BaSnS_2O$ and $SrSnS_2O$ and C_{11} of $CaSnS_2O$) also vary by more than 10 GPa. Accordingly, the precise numerical values of these near-zero coupling terms are interpreted cautiously.

Table S3. Dependence of the calculated elastic stiffness constants C_{ij} (GPa) on the maximum strain amplitude for $ASnS_2O$ (A = Ba, Sr, and Ca).

Elastic stiffness	$BaSnS_2O$	$SrSnS_2O$	$CaSnS_2O$

constant, C_{ij} (GPa)	0.002	0.003	0.004	0.002	0.003	0.004	0.002	0.003	0.004
C_{11}	84.195	85.778	85.381	105.022	107.747	102.782	127.008	115.507	113.167
C_{22}	84.506	98.948	85.305	87.108	69.909	72.411	102.251	86.261	93.279
C_{33}	72.193	73.226	72.987	72.846	68.853	70.790	73.485	74.633	74.352
C_{44}	24.570	25.807	30.342	28.127	28.267	30.588	22.326	25.305	22.374
C_{55}	28.693	28.040	23.740	26.008	27.464	19.653	24.035	26.545	19.914
C_{66}	32.511	30.321	29.126	35.131	34.242	36.121	41.004	38.706	36.458
C_{12}	26.076	30.915	29.093	31.467	31.104	29.778	30.964	23.907	25.798
C_{13}	19.764	19.805	20.836	20.452	20.382	21.388	24.482	19.268	19.614
C_{14}	2.792	4.526	3.007	1.462	3.677	4.165	2.182	5.487	4.067
C_{15}	−0.261	0.079	1.362	1.922	2.907	0.778	1.228	0.918	0.968
C_{16}	2.525	2.305	3.169	−1.489	−0.171	−0.216	0.990	−0.351	1.847
C_{23}	37.400	41.095	39.196	35.332	40.433	36.476	39.924	34.510	34.582
C_{24}	−5.276	0.330	−2.523	−0.308	2.030	0.763	2.674	4.893	4.903
C_{25}	−1.997	−1.855	−1.803	3.832	0.225	1.315	2.416	−1.855	−1.625
C_{26}	5.739	3.473	4.421	1.890	2.055	3.648	9.740	6.635	5.936
C_{34}	−2.846	1.789	−0.426	4.200	−2.177	2.234	−1.788	2.337	−0.552
C_{35}	1.297	2.854	2.132	−1.116	0.625	0.348	−2.655	−1.420	−0.552
C_{36}	−1.049	−1.215	1.910	−1.420	−1.728	−2.589	1.876	−0.157	−0.580
C_{45}	−3.373	−1.999	−1.174	−1.362	−1.300	0.500	0.940	0.948	2.396
C_{46}	1.617	0.653	−1.445	−1.179	−0.122	−0.744	0.096	1.526	−0.242
C_{56}	1.099	2.735	1.493	1.845	2.687	3.122	4.523	3.213	5.758

Note. The maximum strain amplitudes considered are 0.002, 0.003, and 0.004. The 0.003 datasets correspond to the elastic constants reported in Table 3 of the main manuscript.

Table S3b. Voigt–Reuss–Hill bulk modulus (B_H), shear modulus (G_H), Pugh ratio (B/G), universal anisotropy index (A_U) and minimum stiffness-matrix eigenvalue recomputed from the elastic constants of Table S3 at the three maximum strain amplitudes for $ASnS_2O$ (A = Ba, Sr, and Ca).

Max. strain	Compound	B_H (GPa)	G_H (GPa)	B/G	A_U	Min. eigenvalue (GPa)
0.002	$BaSnS_2O$	44.880	26.812	1.674	0.346	21.1
	$SrSnS_2O$	48.208	28.920	1.667	0.304	25.0
	$CaSnS_2O$	53.572	29.466	1.818	0.711	21.6
0.003	$BaSnS_2O$	47.929	27.283	1.757	0.284	23.0
	$SrSnS_2O$	47.413	26.520	1.788	0.737	25.1
	$CaSnS_2O$	47.225	30.121	1.568	0.461	24.2
0.004	$BaSnS_2O$	46.376	26.324	1.762	0.263	22.6
	$SrSnS_2O$	46.433	26.377	1.760	0.598	19.0
	$CaSnS_2O$	48.282	27.349	1.765	0.728	16.6

Note. The 0.003 rows correspond to Table 4 of the main manuscript. All stiffness matrices remain positive definite at the three strain amplitudes (minimum eigenvalues 16.6–25.1 GPa). The highest shear modulus of $CaSnS_2O$ is retained at all three amplitudes, whereas the lowest shear modulus, the highest Pugh ratio and the highest anisotropy index of $SrSnS_2O$, the lowest Pugh ratio of $CaSnS_2O$, and the near invariance of the bulk modulus are most clearly seen at 0.003; the polycrystalline properties derived from the 0.003 dataset are therefore regarded as indicative.

IV. Convergence with respect to k-point sampling and plane-wave cutoff

To assess the numerical sensitivity of the calculated properties, $BaSnS_2O$ was selected as a representative composition. Two sets of convergence tests were performed. First, the plane-wave cutoff energy was varied from 550 to 750 eV while keeping the Monkhorst–Pack k-point mesh fixed at 5×3×2. Second, the k-point mesh was varied from 5×3×2 to 7×3×2 while maintaining the plane-wave cutoff energy at 650 eV. The band-gap energy (Eg), the elastic stiffness constant C_{11}, and the polycrystalline-averaged dielectric function $\square_2(\square)$ were examined. The corresponding Eg and C_{11} values are summarized in Table S4, while the convergence of $\square_2(\square)$ is presented in Figure S1.

Table S4. Convergence of the calculated band gap Eg and elastic stiffness constant C_{11} of $BaSnS_2O$ with respect to the plane-wave cutoff energy and k-point mesh.

Plane-wave cutoff (eV)	k-point mesh	Eg (eV)	C_{11}(GPa)
550	5×3×2	1.460	84.433
650	5×3×2	1.461	85.778
750	5×3×2	1.463	91.090
650	6×3×2	1.461	85.440
650	7×3×2	1.461	82.639

Note. The 650 eV cutoff energy and 5×3×2 k-point mesh correspond to the computational settings employed in the main calculations.

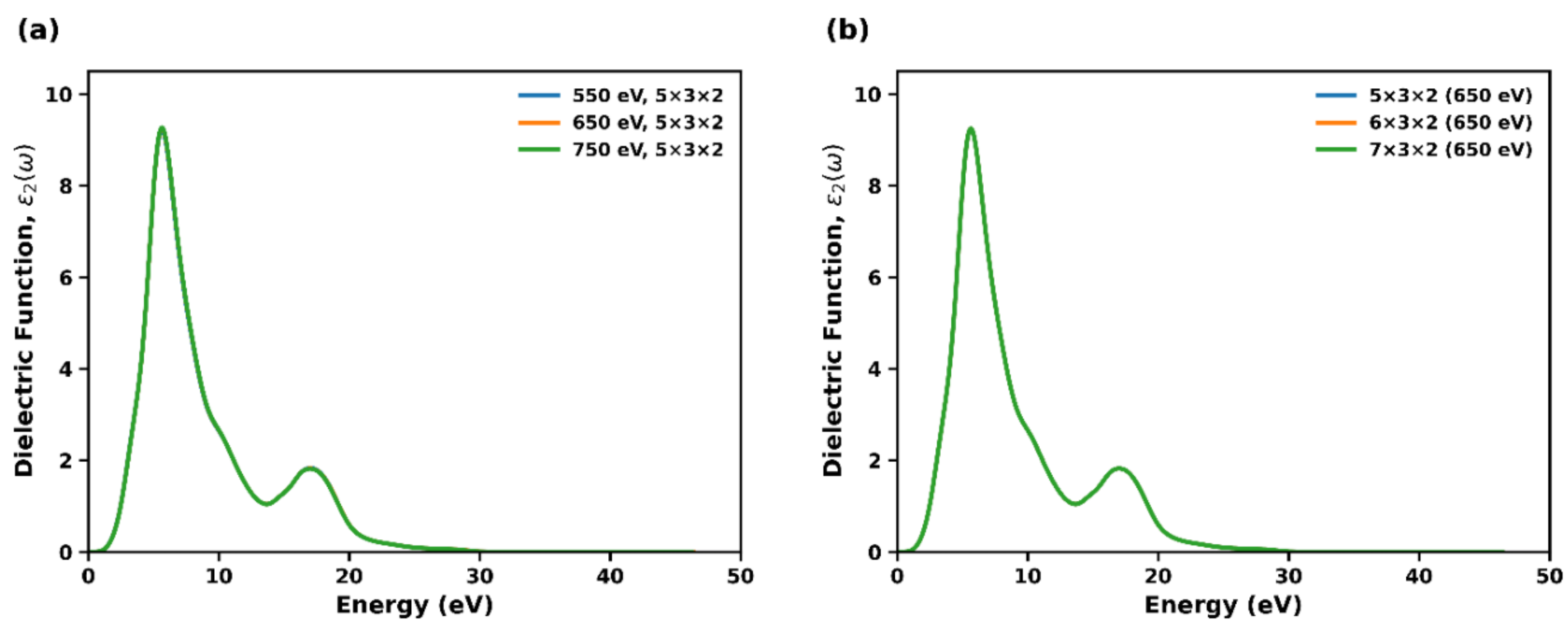

Fig. S1. Convergence of the polycrystalline-averaged dielectric function, $ε_2(ω)$, of $BaSnS_2O$ with respect to **(a)** the plane-wave cutoff energy at a fixed 5×3×2 k-point mesh and **(b)** the k-point mesh at a fixed plane-wave cutoff energy of 650 eV.

The calculated band gap shows very weak dependence on the tested computational parameters, varying only from 1.460 to 1.463 eV over the 550–750 eV cutoff range and remaining at 1.461 eV for all three investigated k-point meshes. The polycrystalline-averaged $ε_2(ω)$ spectra also show close agreement in spectral shape and peak positions for the tested cutoff energies and k-point meshes. The C_{11} values exhibit greater numerical variation, ranging from 82.639 to 91.090 GPa (up to ∼10% of the reference value) over the tested settings. Overall, the convergence tests demonstrate limited sensitivity of the principal electronic and optical results to the investigated computational parameters, while C_{11} shows a numerical dependence of up to ∼10% on the cutoff energy and k-point sampling.

V. Optical Properties: Dielectric Tensor and Polarization-Dependent Response

The frequency-dependent real and imaginary components of the reconstructed complex dielectric tensor for triclinic $ASnS_2O$ (A = Ba, Sr, and Ca) are presented in Figs. S2–S4. Diagonal (xx, yy, zz) and off-diagonal (xy, xz, yz) terms are displayed separately for each composition to illustrate the polarization-dependent dielectric response. The corresponding polycrystalline-averaged optical spectra are presented in Figs. 5–9 and discussed in Section 3.3, "Optical Properties," of the main manuscript.

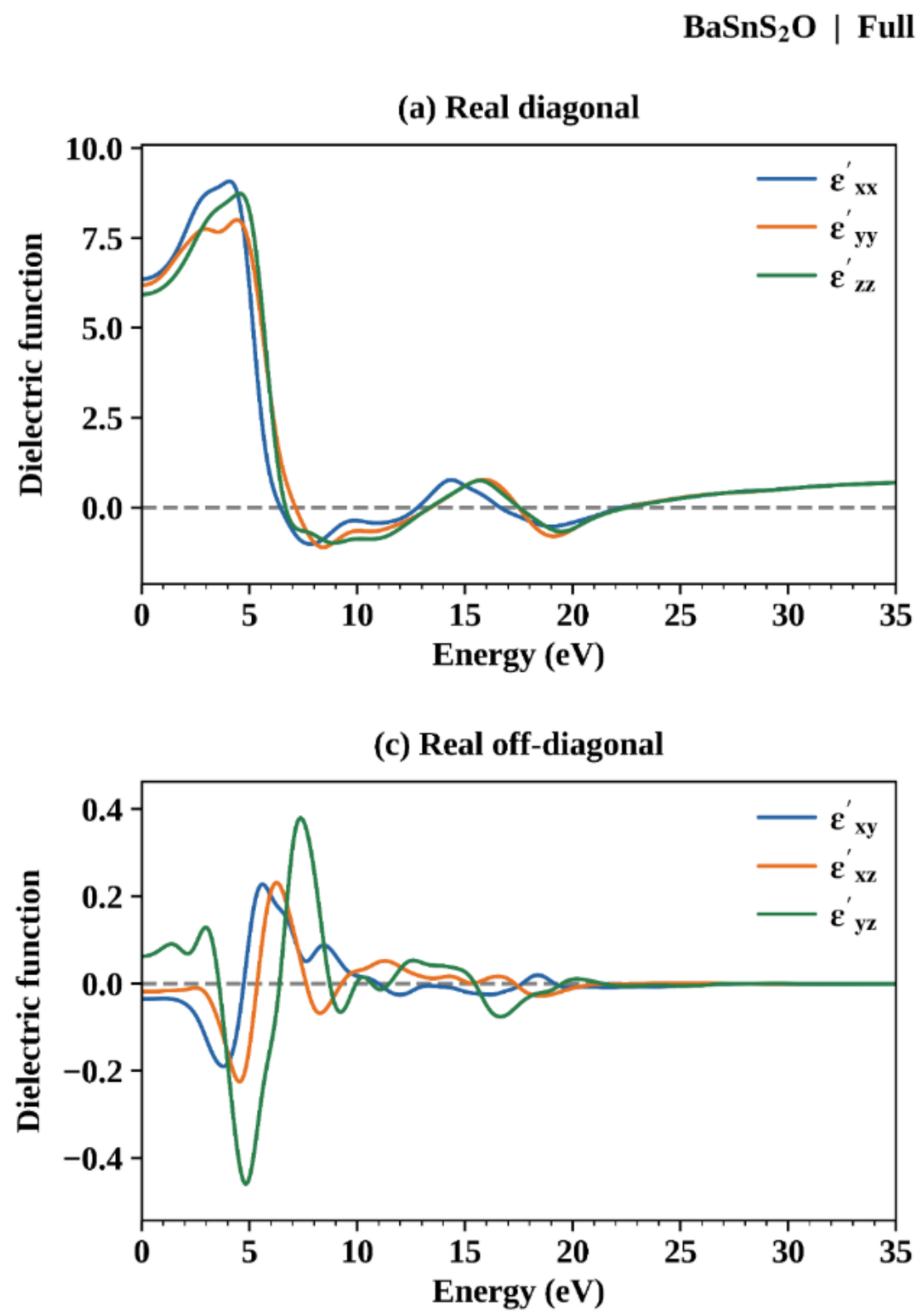

Fig. S2. Frequency-dependent components of the reconstructed complex dielectric tensor of $BaSnS_2O$: (a) real diagonal, (b) imaginary diagonal, (c) real off-diagonal, and (d) imaginary off-diagonal components in the 0–35 eV photon-energy range.

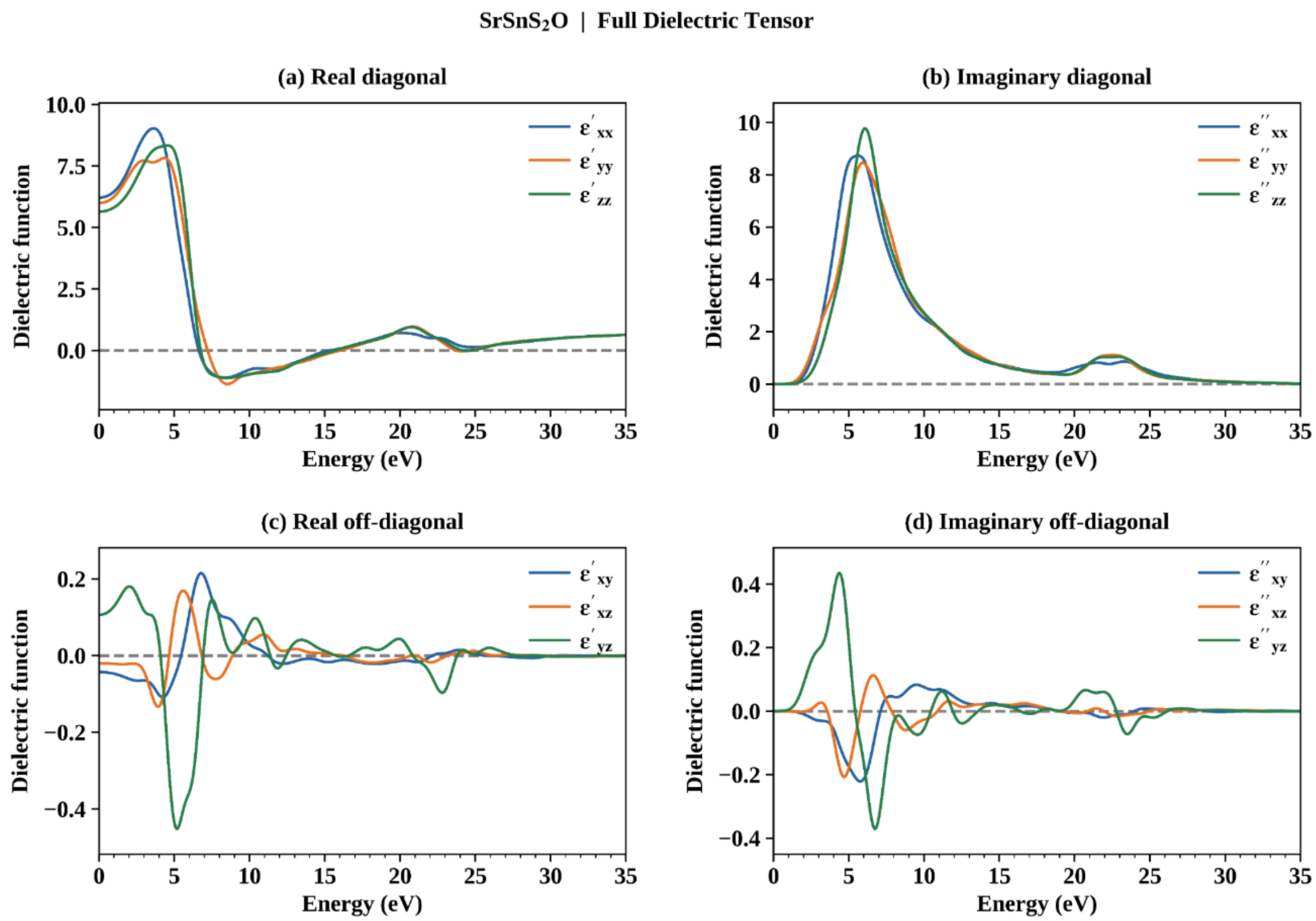

Fig. S3. Frequency-dependent components of the reconstructed complex dielectric tensor of $SrSnS_2O$: (a) real diagonal, (b) imaginary diagonal, (c) real off-diagonal, and (d) imaginary off-diagonal components in the 0–35 eV photon-energy range.

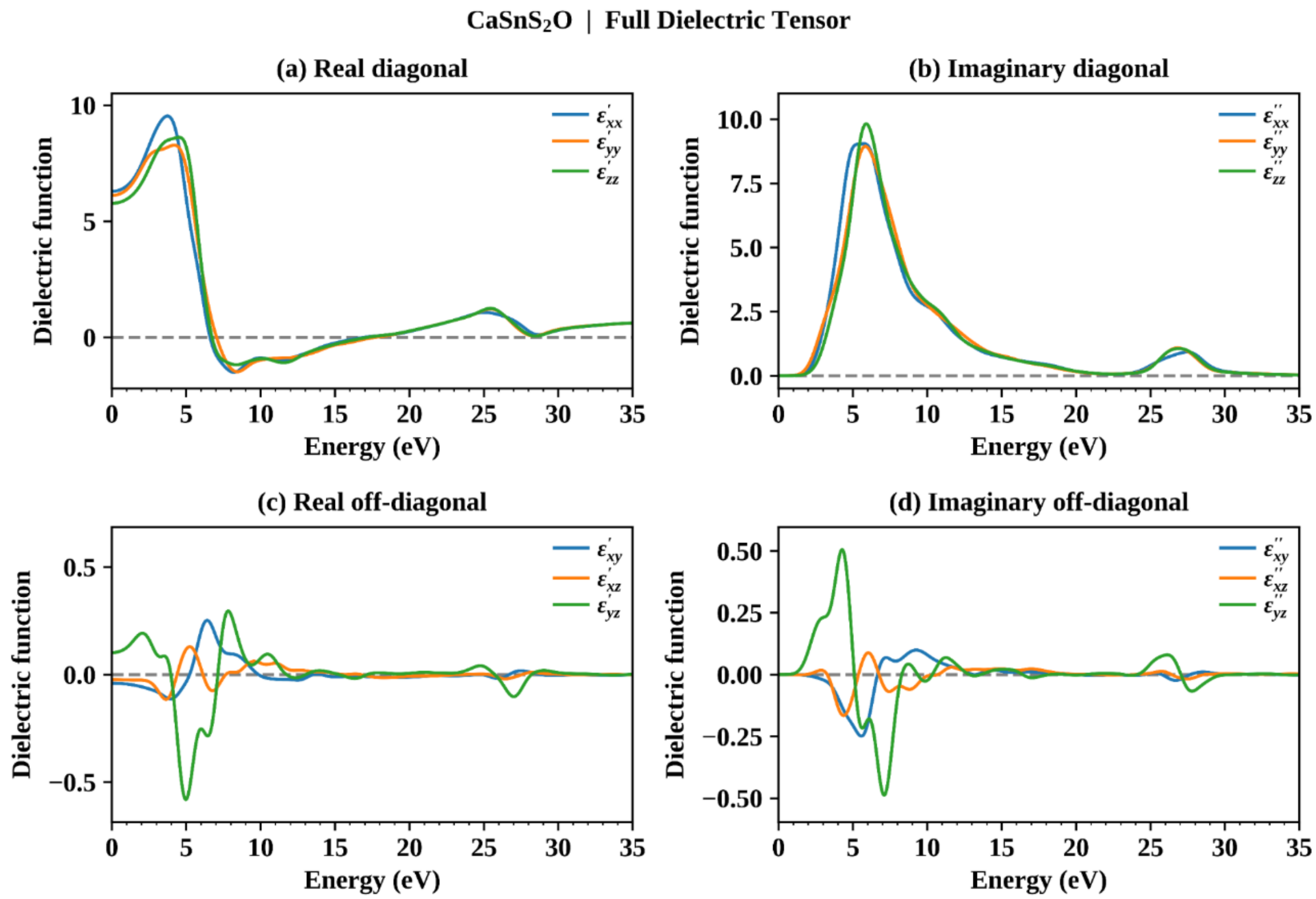

Fig. S4. Frequency-dependent components of the reconstructed complex dielectric tensor of $CaSnS_2O$: (a) real diagonal, (b) imaginary diagonal, (c) real off-diagonal, and (d) imaginary off-diagonal components in the 0–35 eV photon-energy range.

Polarization-dependent optical spectra along (1,0,0)

Figures S5–S9 present the optical spectra calculated for the (1,0,0) polarization direction of $BaSnS_2O$, $SrSnS_2O$, and $CaSnS_2O$. These plots document the direction-specific response of the triclinic structures and are retained to complement the dielectric-tensor components in Figures S2–S4. Because a single polarization direction cannot fully represent the overall optical behavior, the main manuscript uses polycrystalline-averaged optical spectra in Figs. 5–9 for comparison among the three compositions.

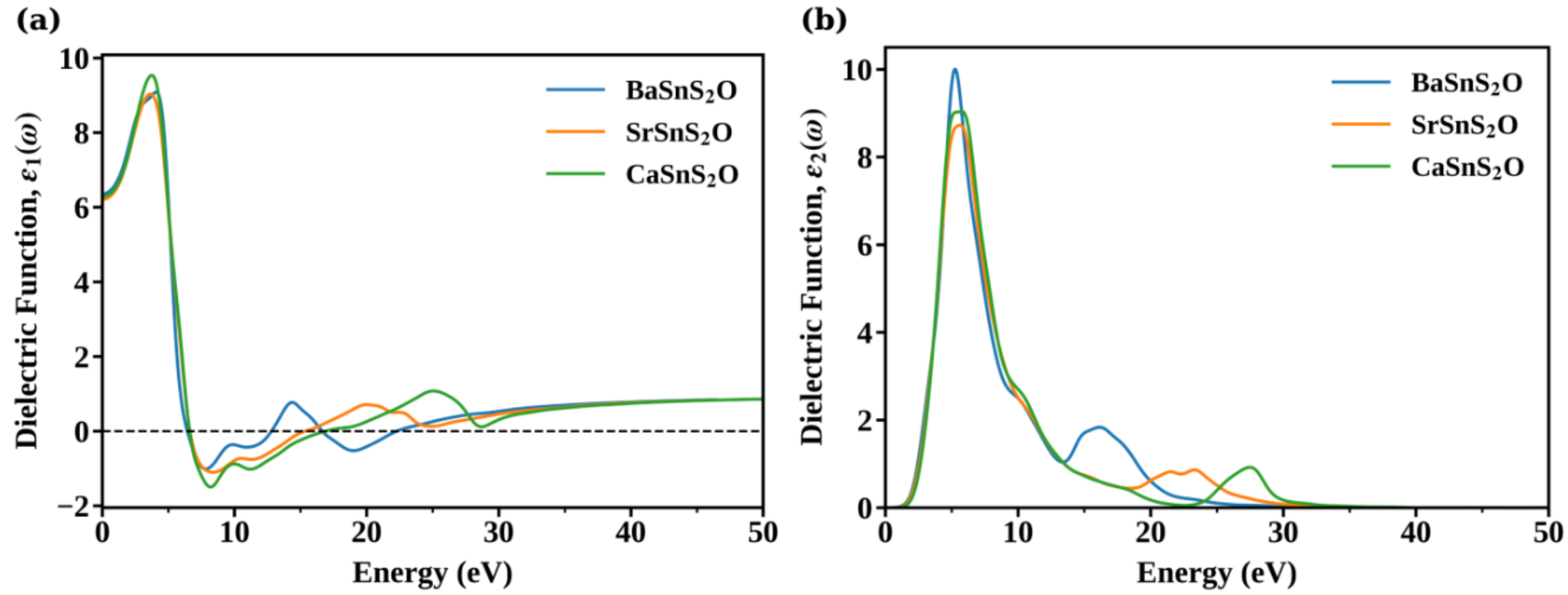

Fig. S5. Calculated (a) real, $\square_1(\square)$, and (b) imaginary, $\square_2(\square)$, dielectric functions of $BaSnS_2O$, $SrSnS_2O$, and $CaSnS_2O$ for the (1,0,0) polarization direction over the 0–50 eV photon-energy range.

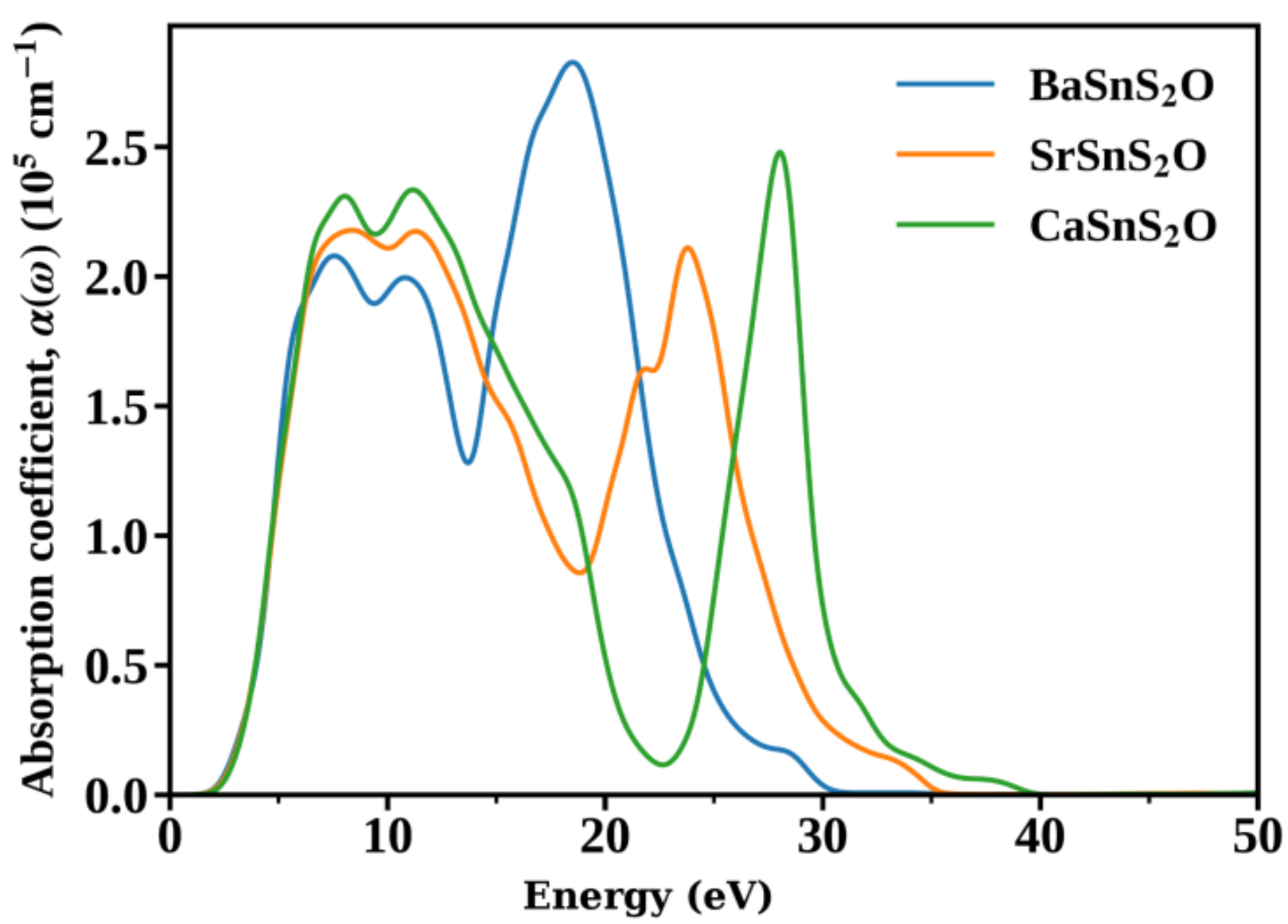

Fig. S6. Calculated absorption coefficients, $\square(\square)$, of $BaSnS_2O$, $SrSnS_2O$, and $CaSnS_2O$ for the (1,0,0) polarization direction as functions of photon energy (0–50 eV).

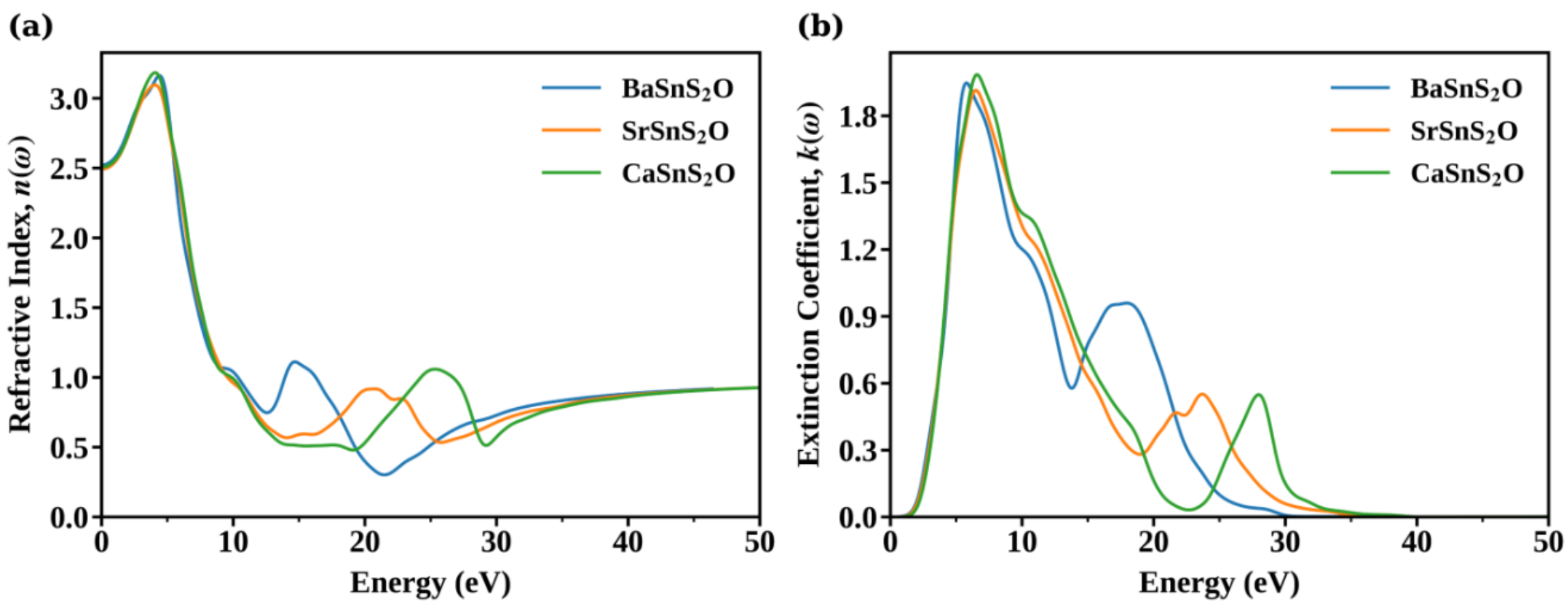

Fig. S7. Calculated (a) refractive index, n(ω), and (b) extinction coefficient, k(ω), of $BaSnS_2O$, $SrSnS_2O$, and $CaSnS_2O$ for the (1,0,0) polarization direction (0–50 eV).

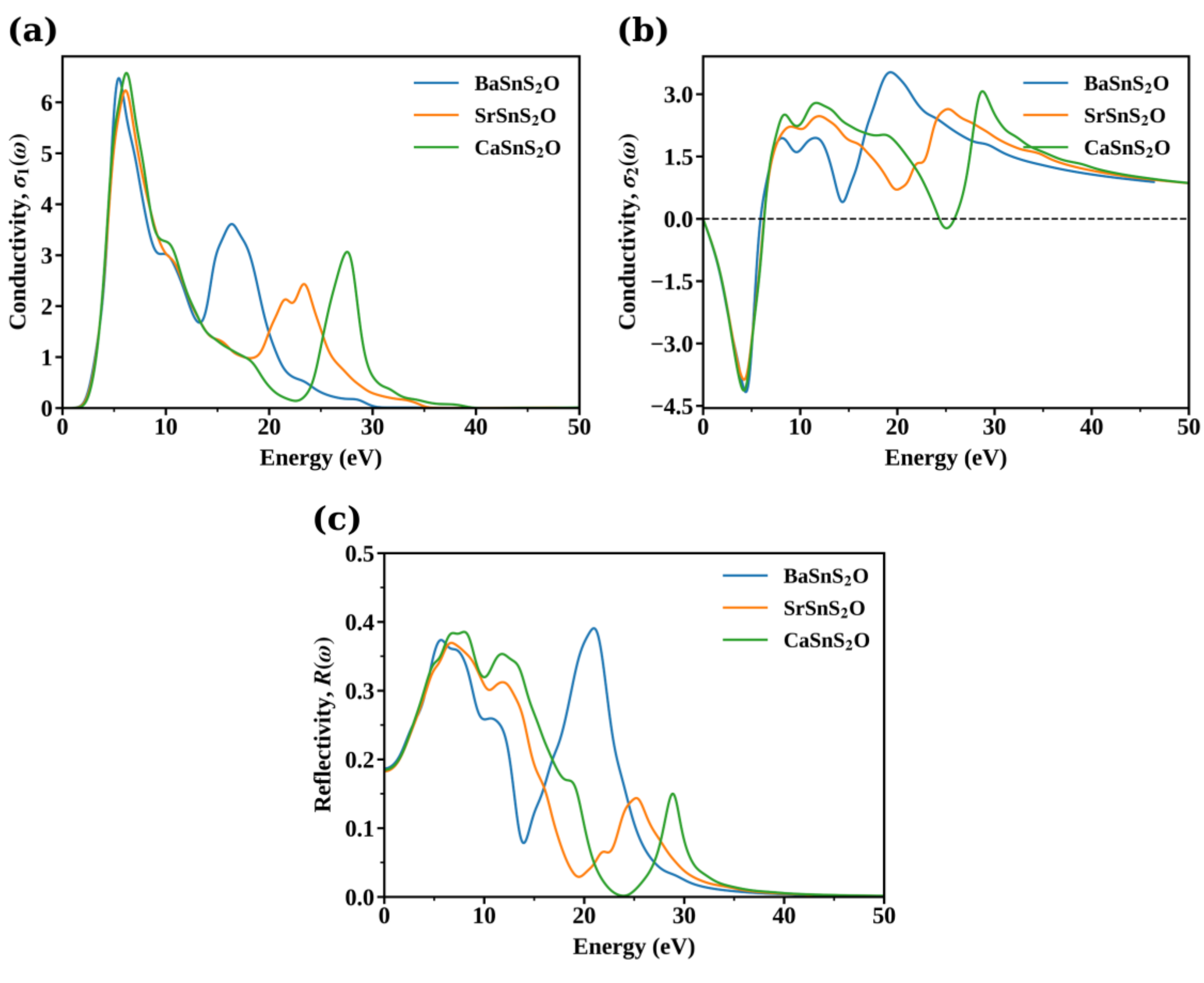

Fig. S8. Calculated (a) real optical conductivity, σ$_1$(ω), (b) imaginary optical conductivity, σ$_2$(ω), and (c) reflectivity, R(ω), of $BaSnS_2O$, $SrSnS_2O$, and $CaSnS_2O$ for the (1,0,0) polarization direction (0–50 eV).

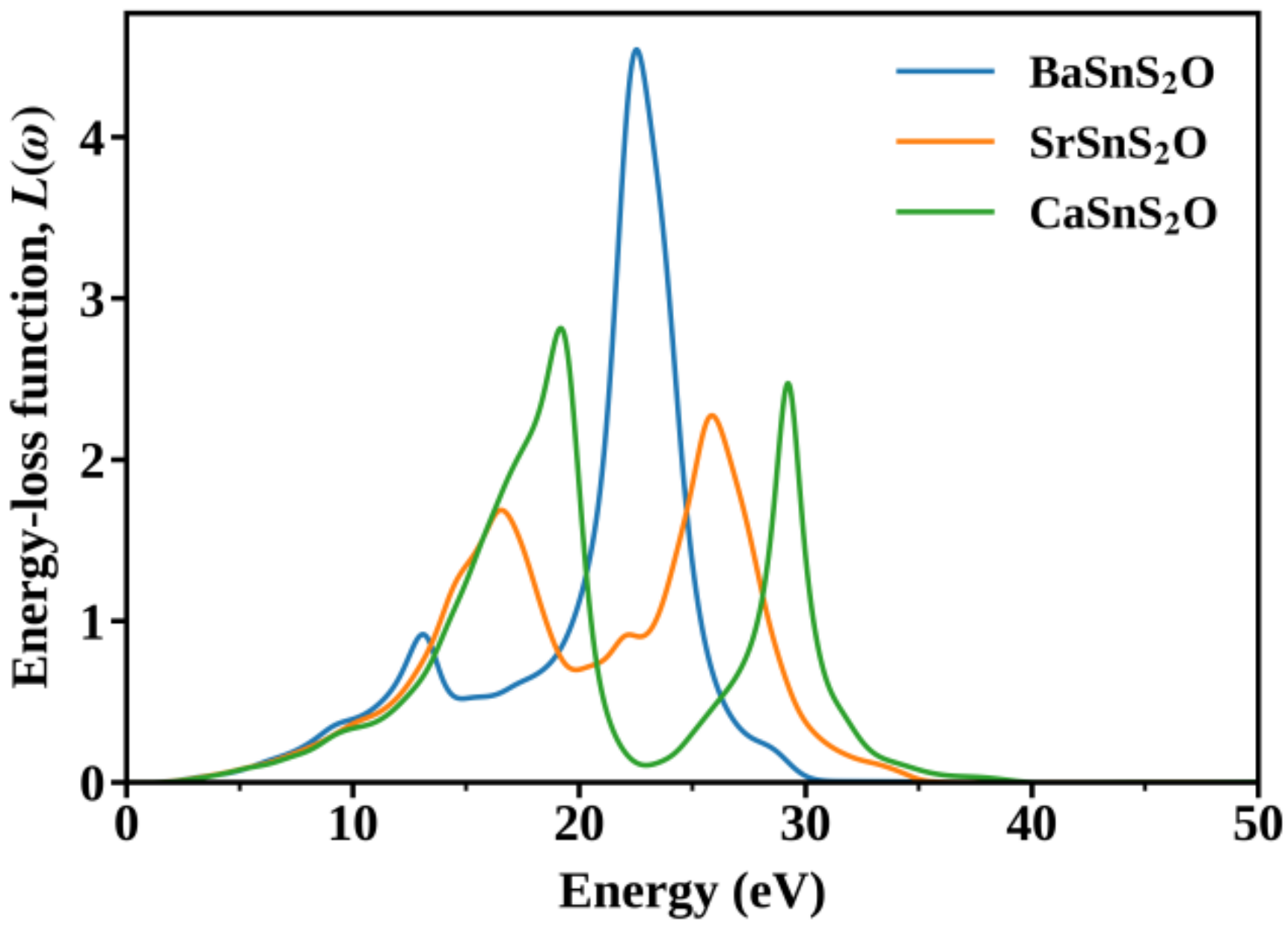

Fig. S9. Calculated energy-loss function, L(ω), of $BaSnS_2O$, $SrSnS_2O$, and $CaSnS_2O$ for the (1,0,0) polarization direction as a function of photon energy (0–50 eV).

VI. Band-edge dispersion and CASTEP Mulliken populations

CASTEP Mulliken bond populations and the sampled G–F–Q–Z–G widths of the highest occupied and lowest unoccupied bands are summarized in Table S5. The populations represent total Sn–anion atom-pair overlaps, not orbital-pair-resolved interactions.

Table S5. CASTEP-reported Mulliken bond overlap populations and sampled-path frontier-band widths of fully relaxed $ASnS_2O$ (A = Ba, Sr, Ca).

Compound	⟨P(Sn–S)⟩	P(Sn–S) range	⟨P(Sn–O)⟩	P(Sn–O) range	W_{VB} (eV)	W_{CB} (eV)
BaSnS₂O	0.655	0.33–0.99	0.502	0.39–0.61	0.01064	0.11483
SrSnS₂O	0.639	0.29–0.96	0.522	0.38–0.67	0.14155	0.09041
CaSnS₂O	0.640	0.27–0.97	0.512	0.33–0.67	0.15674	0.01967

Note. Each Mulliken mean/range covers the 11 Sn–S or 5 Sn–O pair entries reported by CASTEP, rather than all 17 periodic first-shell Sn–S contacts in main-text Table 2. W_{VB} and W_{CB} are the sampled-path widths of bands 64 and 65 (13 k points for Ba, 15 for Sr/Ca), not full-Brillouin-zone bandwidths.

Although the gap rises 1.461 → 1.727 → 1.928 eV from Ba to Ca, the mean Sn–S Mulliken overlap does not increase (0.655 → 0.639 → 0.640). W_{VB} increases whereas W_{CB} decreases. These trends do not support a simple uniform bond-shortening/overlap explanation.

VII. Reciprocal fixed-volume and local-geometry analysis

Reciprocal fixed-volume calculations test the effect of changing lattice volume while retaining each A-site composition. Table S6 compares the resulting gaps and local structures. Sn–S mean distances include all 17 periodic first-shell contacts per 20-atom cell.

Table S6. Equilibrium and reciprocal fixed-volume comparisons of band gaps and local Sn–anion geometry.

Calculation	V (Å^3)	Eg (eV)	Mean Sn–S (Å)	Sn–S range (Å)	O–Sn–O range (°)
$BaSnS_2O$, equilibrium	446.001	1.461	2.564	2.375–2.854	104.23–162.36
$SrSnS_2O$, equilibrium	418.804	1.727	2.556	2.395–2.788	99.66–169.41
$CaSnS_2O$, equilibrium	394.341	1.928	2.536	2.408–2.689	96.78–174.78
$BaSnS_2O$ at V_{Ca}	394.340	1.389	2.508	2.341–2.703	103.97–165.98
$CaSnS_2O$ at V_{Ba}	445.996	1.795	2.576	2.434–2.740	92.45–176.80

Note. V_{Ca} and V_{Ba} are the equilibrium Ca and Ba cell volumes. The cross-volume structures were relaxed internally at fixed lattice. Their gaps (1.389 and 1.795 eV) were calculated in this work. Angular ranges describe heterogeneity, not a uniquely isolated band-gap contribution.

Compressing $BaSnS_2O$ to V_{Ca} reduces Eg by 0.072 eV, whereas expanding $CaSnS_2O$ to V_{Ba} reduces Eg by 0.133 eV. At nearly matched volumes, the Ca–Ba gap differences remain 0.539 eV (V_{Ca}) and 0.334 eV (V_{Ba}). Thus, the increasing equilibrium gap cannot be explained by volume alone. Because A-site identity, lattice shape, and relaxed local geometry also differ, these comparisons do not separately quantify chemical and angular-distortion effects.

VIII. Sn-5p DFT+U (PBE+U) band-gap and water-redox screening sensitivity

To assess the sensitivity of the calculated band gaps to Hubbard corrections, additional DFT+U electronic band-structure calculations were performed within the GGA-PBE framework (hereafter PBE+U) using the previously PBE-optimized $BaSnS_2O$, $SrSnS_2O$, and $CaSnS_2O$ structures. The PBE-optimized geometries were retained without further structural relaxation, and Hubbard U values of 2, 3, and 4 eV were applied to the Sn-5p subspace of each compound. The selection of this subspace was guided by the orbital characteristics identified in the

present projected density of states (PDOS) analysis and by previously published DFT+U investigations of Sn-containing semiconductors.

As discussed in Section 3.2.2 and illustrated in Fig. 4 of the main manuscript, the electronic states near the valence-band edge are predominantly derived from S-3p and O-2p orbitals, whereas the conduction-band edge exhibits mainly Sn-derived s/p contributions. Based on this orbital distribution, the Sn-5p subspace was selected as a band-edge-related component for evaluating the sensitivity of the electronic band gaps to Hubbard corrections. However, the observed PDOS contributions do not establish that the Sn-5p states independently determine the band gap or that applying a Hubbard correction exclusively to this subspace provides quantitatively accurate band-gap predictions.

The methodological basis for examining Sn-p Hubbard corrections is further supported by previous first-principles studies. Kirchner-Hall et al.[2] included Sn-5p Hubbard subspaces in their DFT+U benchmarking of SnS_2 and SnO_2, reporting U values of 1.37 and 1.26 eV, respectively, within their specific Hubbard-projector implementation, while also considering corrections to anion-p states. Similarly, Babuka et al. [3] investigated p-orbital Hubbard corrections in Sn₂P_2S_6 using CASTEP, including calculations with Sn-p-only corrections. Their results indicated that corrections applied to S- and P-p states had a greater influence than the Sn-p-only correction in that compound. These investigations provide methodological precedent for examining Hubbard corrections to Sn-p states; however, they do not establish the quantitative accuracy of Sn-5p-only corrections or validate specific U values for the present $ASnS_2O$ compounds. Given the dependence of Hubbard U on the local chemical environment and computational projector scheme, the U = 2–4 eV range adopted in this work is intended as an exploratory sensitivity assessment rather than a first-principles determination, empirical calibration, or demonstration of improved absolute band-gap accuracy.

Table S7. Comparison of PBE and Sn-5p PBE+U electronic band gaps calculated using the same PBE-relaxed $ASnS_2O$ structures.

Compound	PBE<br>Eg (eV)	PBE+U<br>U = 2 eV	PBE+U<br>U = 3 eV	PBE+U<br>U = 4 eV
$BaSnS_2O$	1.461	1.497	1.517	1.537
$SrSnS_2O$	1.727	1.788	1.821	1.854
$CaSnS_2O$	1.928	1.992	2.025	2.058

Note. The PBE+U results are fixed-PBE-geometry electronic calculations, not fully PBE+U-relaxed calculations. The investigated Sn-5p U values are exploratory sensitivity parameters and are not claimed to be experimentally calibrated or uniquely optimal.

For all three compositions, the gap increases with U across the tested 2–4 eV range, while the ordering $Eg(BaSnS_2O) < Eg(SrSnS_2O) < Eg(CaSnS_2O)$ remains unchanged. Relative to PBE, the U = 4 eV gaps increase by 0.076, 0.127, and 0.130 eV for $BaSnS_2O$, $SrSnS_2O$, and $CaSnS_2O$, respectively. Thus, the relative band-gap

ordering is stable over the tested Sn-5p U range; this does not independently establish the quantitative accuracy of the absolute gaps.

To assess how these gap changes propagate through the water-redox screening approach used in the main manuscript, the PBE and PBE+U gaps were inserted into the same Mulliken electronegativity-based expressions, ECB = χ − 4.50 − Eg/2 and EVB = χ − 4.50 + Eg/2 (in V versus NHE). The compound electronegativities were kept fixed at χ = 5.055, 5.127, and 5.195 eV for $BaSnS_2O$, $SrSnS_2O$, and $CaSnS_2O$, respectively. Table S8 shows these calculated screening estimates at pH = 0; they are not independently computed absolute band-edge positions from a PBE+U interface or vacuum alignment.

Table S8. Illustrative pH = 0 water-redox screening estimates obtained by inserting PBE and Sn-5p PBE+U gaps into the main-text electronegativity model.

Compound	U on Sn-5p (eV)	Eg (eV)	ECB (V vs NHE)	EVB (V vs NHE)	HER offset (V)	OER offset (V)
$BaSnS_2O$	0 (PBE)	1.461	-0.1755	+1.2855	+0.1755	+0.0555
$BaSnS_2O$	2	1.497	-0.1935	+1.3035	+0.1935	+0.0735
$BaSnS_2O$	3	1.517	-0.2035	+1.3135	+0.2035	+0.0835
$BaSnS_2O$	4	1.537	-0.2135	+1.3235	+0.2135	+0.0935
$SrSnS_2O$	0 (PBE)	1.727	-0.2365	+1.4905	+0.2365	+0.2605
$SrSnS_2O$	2	1.788	-0.2670	+1.5210	+0.2670	+0.2910
$SrSnS_2O$	3	1.821	-0.2835	+1.5375	+0.2835	+0.3075
$SrSnS_2O$	4	1.854	-0.3000	+1.5540	+0.3000	+0.3240
$CaSnS_2O$	0 (PBE)	1.928	-0.2690	+1.6590	+0.2690	+0.4290
$CaSnS_2O$	2	1.992	-0.3010	+1.6910	+0.3010	+0.4610
$CaSnS_2O$	3	2.025	-0.3175	+1.7075	+0.3175	+0.4775
$CaSnS_2O$	4	2.058	-0.3340	+1.7240	+0.3340	+0.4940

Note. At pH = 0, the H^+/H_2 and O_2/H_2O reference potentials are 0 and +1.23 V vs NHE, respectively. The nominal HER offset is $0 - E_{CB}$, and the nominal OER offset is $E_{VB} - 1.23$ V. Positive values indicate nominal thermodynamic alignment, not catalytic overpotentials. Four decimal places aid arithmetic reproducibility, not physical precision; main-text PBE estimates are rounded to three decimals.

At all tested U values, nominal CBM values remain negative relative to H^+/H_2 (0 V vs NHE), and nominal VBM values remain above O_2/H_2O (+1.23 V), giving positive HER and OER offsets. The nominal HER offsets span

0.176–0.214 V ($BaSnS_2O$), 0.237–0.300 V ($SrSnS_2O$), and 0.269–0.334 V ($CaSnS_2O$); $BaSnS_2O$ retains a small OER offset of 0.056–0.094 V. However, these changes follow mathematically from increasing Eg at fixed χ and do not independently validate actual absolute band-edge shifts. Uncertainties in the Hubbard U choice and in the electronegativity model—particularly for borderline $BaSnS_2O$—remain significant. Accordingly, the results show qualitative screening sensitivity only, not practical water-splitting feasibility, quantitative HER/OER driving forces, or a uniquely optimal U.

IX. S/O Configuration Enumeration and Screening

For each of the $BaSnS_3$- and $SrSnS_3$-derived parent frameworks, four of the twelve sulfur sites were selected for oxygen substitution, yielding C (12,4) = 495 possible substitution patterns before symmetry reduction. Symmetry-equivalent arrangements were identified and removed using a symmetry-based enumeration approach implemented in pymatgen, resulting in 135 symmetry-distinct configurations for each parent framework. A summary of the enumeration and screening procedure is presented in Table S9.

The symmetry-distinct configurations were screened using the Forcite module in Materials Studio 2020 with the Universal force field and Smart geometry-optimization algorithm. During screening, the unit-cell parameters were fixed at their respective initial values. The five configurations with the lowest Forcite screening energies for each of $BaSnS_2O$ and $SrSnS_2O$ were selected for subsequent CASTEP geometry optimization and electronic band-gap calculations. The Forcite screening energies of the selected configurations are presented in Table S10, while their CASTEP total energies, relative energies, and band gaps are summarized in Table S11.

The BaSnS₂O, $SrSnS_2O$, and $CaSnS_2O$ structures originally investigated in the main manuscript share the same ordered S/O substitution motif, allowing a controlled comparison of A-site substitution effects. In contrast, the additional $BaSnS_2O$ and $SrSnS_2O$ configurations were selected independently based on their respective Forcite screening energies and therefore do not necessarily exhibit identical S/O ordering patterns. These additional calculations were performed to assess the influence of S/O configurational variations within each composition.

For $CaSnS_2O$, five S/O ordering patterns corresponding to the selected $SrSnS_2O$ configurations were applied to the $SrSnS_3$-derived parent framework by replacing Sr with Ca before CASTEP geometry optimization. The previously relaxed $SrSnS_2O$ structures were not used as starting configurations. Since independent symmetry enumeration and Forcite screening were not performed for $CaSnS_2O$, these five configurations are not claimed to represent its five lowest-energy arrangements.

Table S9. Summary of S/O configuration enumeration and screening for $ASnS_2O$ (A = Ba, Sr, Ca).

Parameter	$BaSnS_2O$	$SrSnS_2O$	$CaSnS_2O$
Possible arrangements before symmetry reduction	495	495	495*

Parameter	$BaSnS_2O$	$SrSnS_2O$	$CaSnS_2O$
Symmetry-distinct configurations enumerated	135	135	Not independently enumerated
Configurations screened using force-field energies	135	135	Not independently screened
Candidates selected for CASTEP follow-up	5	5	5 corresponding Sr-selected orderings

*For $CaSnS_2O$, 495 represents the combinatorial number of possible substitution patterns. Independent symmetry reduction and Forcite screening were not performed; the five CASTEP configurations were constructed using the selected $SrSnS_2O$ ordering patterns.

Table S9a. Initial oxygen fractional coordinates in the primary $ASnS_2O$ configurations.

O position	$BaSnS_2O$ (x, y, z)	$SrSnS_2O$ (x, y, z)	$CaSnS_2O$ (x, y, z)
O1	(0.75000, 0.33671, 0.50235)	(0.75000, 0.34730, 0.50441)	(0.75000, 0.34730, 0.50441)
O2	(0.75000, 0.97250, 0.40020)	(0.75000, 0.97278, 0.39567)	(0.75000, 0.97278, 0.39567)
O3	(0.75000, 0.71257, 0.70785)	(0.75000, 0.70193, 0.71453)	(0.75000, 0.70193, 0.71453)
O4	(0.25000, 0.66329, 0.49765)	(0.25000, 0.65270, 0.49559)	(0.25000, 0.65270, 0.49559)

Note: The fractional coordinates correspond to the initial structures before geometry optimization. Although $BaSnS_2O$ and $SrSnS_2O$ were constructed from separate parent structures, oxygen substitution was performed at corresponding anion positions to maintain a common initial S/O ordering motif. The initial oxygen positions in $CaSnS_2O$ are identical to those in $SrSnS_2O$, with Sr replaced by Ca. O1–O4 denote corresponding oxygen positions for comparison

Table S10. Forcite screening energies and oxygen-substituted sulfur sites of the selected $BaSnS_2O$ and $SrSnS_2O$ configurations.

Compound	Configuration ID	Forcite screening energy (kcal mol^{-1})	O-substituted S sites
$BaSnS_2O$	59	88.677506	S8, S10, S17, S19
$BaSnS_2O$	46	93.806331	S8, S10, S13, S19
$BaSnS_2O$	91	95.620592	S8, S12, S14, S16
$BaSnS_2O$	71	96.867291	S8, S11, S13, S18

Compound	Configuration ID	Forcite screening energy (kcal mol^{-1})	O-substituted S sites
$BaSnS_2O$	130	97.146193	S10, S13, S15, S16
$SrSnS_2O$	25	169.074042	S8, S9, S18, S19
$SrSnS_2O$	109	172.661495	S8, S15, S18, S19
$SrSnS_2O$	99	172.850220	S8, S14, S16, S18
$SrSnS_2O$	91	174.906606	S8, S13, S17, S18
$SrSnS_2O$	105	176.786216	S8, S15, S16, S18

Note: Forcite screening energies were used to select candidate configurations within each composition. They are not directly comparable to CASTEP total energies and do not establish the DFT configurational ground state. The O-substituted S sites refer to the original sulfur-site numbering in the respective parent structures. The five $CaSnS_2O$ configurations were constructed using the same S/O substitution patterns as the corresponding $SrSnS_2O$ configurations (IDs 25, 109, 99, 91, and 105), with Sr replaced by Ca. Independent Forcite screening was not performed for $CaSnS_2O$. The corresponding starting CIF files for all 15 configurations are provided in the accompanying Supplementary Data archive.

Table S11. CASTEP total energies, relative energies, and electronic band gaps of the selected $ASnS_2O$ configurations.

Compound	Configuration ID	CASTEP total energy (eV/cell)	Relative energy (eV/cell)	Band gap (eV)
$BaSnS_2O$	59	−7129.2990	0.0000	1.277
$BaSnS_2O$	46	−7128.7213	0.5777	1.470
$BaSnS_2O$	130	−7127.4209	1.8781	1.865
$BaSnS_2O$	91	−7127.1090	2.1900	1.626
$BaSnS_2O$	71	−7126.0481	3.2509	0.920
$SrSnS_2O$	109	−7703.5804	0.0000	2.099
$SrSnS_2O$	25	−7703.4783	0.1021	1.924
$SrSnS_2O$	105	−7703.3650	0.2154	1.910
$SrSnS_2O$	99	−7703.3006	0.2798	1.510

Compound	Configuration ID	CASTEP total energy (eV/cell)	Relative energy (eV/cell)	Band gap (eV)
$SrSnS_2O$	91	−7702.9507	0.6297	1.405
$CaSnS_2O$	91	−8340.4227	0.0000	1.632
$CaSnS_2O$	109	−8340.0871	0.3356	2.026
$CaSnS_2O$	25	−8340.0152	0.4075	1.397
$CaSnS_2O$	99	−8339.7028	0.7199	1.550
$CaSnS_2O$	105	−8339.6811	0.7416	1.916

Note: Relative energies were calculated separately for each composition as ΔEᵢ = Eᵢ – Eₘᵢₙ, where Eₘᵢₙ is the lowest CASTEP total energy among the five evaluated configurations of that composition. All reported CASTEP total and relative energies refer to the 20-atom computational cell. The relative energies therefore establish the energetic ordering only within the selected subsets and do not identify the global configurational ground states. The $CaSnS_2O$ configuration IDs refer to the S/O ordering patterns inherited from the selected $SrSnS_2O$ configurations.

X. Comparison of $CaSnS_2O$ Configurations Derived from Different Parent Frameworks

To address the framework dependence of $CaSnS_2O$, an alternative configuration was constructed by replacing four of the twelve S atoms with O in the Pnma $CaSnS_3$ structure reported in [4]. The resulting structure was fully optimized using CASTEP and compared with the original $SrSnS_3$-derived $CaSnS_2O$ configuration. The optimized structural parameters and total energies are presented in Table S12.

Table S12. Structural and energetic comparison of $CaSnS_2O$ configurations derived from different parent frameworks.

Parameter	$CaSnS_2O$ ($SrSnS_3$-derived)	$CaSnS_2O$ (Pnma-$CaSnS_3$-derived)
a (Å)	3.7006	7.0736
b (Å)	8.4716	6.9755
c (Å)	12.7294	7.5840
α, β, γ (°)	81.186, 90.451, 89.771	90.003, 90.036, 89.994

Parameter	$CaSnS_2O$ ($SrSnS_3$-derived)	$CaSnS_2O$ (Pnma-$CaSnS_3$-derived)
Volume (Å^3)	394.341	374.207
Total energy (eV/cell)	−8339.507607	−8339.433749
ΔE (meV/f.u.)	0.00	+18.46

Note: Both cells contain four formula units. Pnma refers to the parent $CaSnS_3$ framework.

The Pnma-CaSnS₃-derived configuration exhibits a 5.1% smaller unit-cell volume but a higher total energy by 18.46 meV per formula unit. Therefore, the original $SrSnS_3$-derived configuration is energetically preferred between these two structures. This comparison is limited to the investigated configurations and does not establish the global ground-state structure of $CaSnS_2O$. Because the two configurations originate from crystallographically distinct parent frameworks, identical S/O ordering was not imposed. Accordingly, the calculated energy difference reflects the combined effects of structural framework and anion arrangement rather than an isolated framework effect.

XI. Structural Sources and Computational Details of Reference Phases

The initial crystal structures of the elemental references and selected competing phases were obtained from the Materials Project database[5, 6]. These structures were chosen as explicitly identifiable crystallographic models of the elemental constituents and chemically relevant compounds involved in the considered decomposition pathways. Established structural prototypes were adopted where applicable, including rock-salt alkaline-earth oxides and sulfides, layered SnS_2, and rutile-type SnO_2. Specific Materials Project entries were retained for the elemental and ternary-compound polymorphs to ensure reproducibility of the phase-dependent energetic comparisons. The corresponding Materials Project identifiers and crystallographic space groups are listed in Table S13. Because alternative polymorphs were not systematically evaluated, the present results refer to the specified reference phases and do not constitute an exhaustive polymorph search or complete convex-hull analysis. The Pnma $CaSnS_3$ framework employed to construct the alternative $CaSnS_2O$ configuration in Section X is distinct from the selected I4₁/a $CaSnS_3$ reference (mp-1100381) used in the decomposition-energy calculations.

All crystalline reference structures were geometry-optimized using CASTEP within the GGA–PBE framework, with norm-conserving pseudopotentials, a plane-wave cutoff of 650 eV, and phase-specific Monkhorst–Pack k-point meshes. The O_2 molecular reference was constructed separately using an experimental initial O–O distance of approximately 1.21 Å [7] in an approximately 15 × 15 × 15 Å^3 periodic cell, followed by spin-polarized geometry optimization with a net spin of 2. The final CASTEP total energies were normalized per atom for elemental solids, per molecule for O_2, and per formula unit for the binary and ternary compounds. These reference energies were used to calculate the formation and selected-pathway decomposition energies reported in Section 3.5.1 of the main manuscript.

Table S13. Structural sources, crystallographic symmetries, computational parameters, and CASTEP reference energies used in the formation- and decomposition-energy calculations.

Material	MP ID / source	Space group	k-point mesh	Atoms/cell	Energy (eV)
Ba	mp-122	Im-3m (229)	7 × 7 × 7	1	−694.121513
Sr	mp-76	Fm-3m (225)	6 × 6 × 6	1	−837.866441
Ca	mp-45	Fm-3m (225)	4 × 4 × 4	1	−996.999524
Sn	mp-84	I4₁/amd (141)	4 × 4 × 7	2	−95.117628
S	mp-77	Fddd (70)	2 × 2 × 1	32	−277.718729
O_2	Experimental	—	3 × 3 × 3	2	−858.173018
BaO	mp-1342	Fm-3m (225)	7 × 7 × 7	2	−1127.913202
SrO	mp-2472	Fm-3m (225)	7 × 7 × 7	2	−1272.317810
CaO	mp-2605	Fm-3m (225)	7 × 7 × 7	2	−1431.836829
BaS	mp-1500	Fm-3m (225)	5 × 5 × 5	2	−975.969767
SrS	mp-1087	Fm-3m (225)	7 × 7 × 7	2	−1119.997753
CaS	mp-1672	Fm-3m (225)	5 × 5 × 5	2	−1279.104514

Material	MP ID / source	Space group	k-point mesh	Atoms/cell	Energy (eV)
SnS_2	mp-1170	P-3m1 (164)	8 × 8 × 7	3	−652.742372
SnO_2	mp-856	P4₂/mnm (136)	7 × 7 × 7	6	−961.270715
$BaSnS_3$	mp-1183370	Pnma (62)	4 × 5 × 2	20	−1628.794291
$SrSnS_3$	mp-1205419	Pnma (62)	3 × 6 × 2	20	−1772.654725
$CaSnS_3$	mp-1100381	I4₁/a (88)	2 × 2 × 2	80	−1931.468793
$BaSnO_3$	mp-3163	Pm-3m (221)	6 × 6 × 6	5	−2089.870091
$SrSnO_3$	mp-2879	Pnma (62)	4 × 4 × 4	20	−2233.920712
$CaSnO_3$	mp-4438	Pnma (62)	4 × 4 × 4	20	−2393.518556

Note: The MP IDs specify the initial crystalline models; the energies listed here were independently calculated using CASTEP, not taken from Materials Project. Energies are given per atom for the elemental solids (Ba, Sr, Ca, Sn, and S), per O_2 molecule, and per formula unit for binary and ternary compounds. Atoms/cell refers to the computational cell. No lowest-energy-polymorph assignment or complete thermodynamic convex-hull analysis is implied by these selected-phase comparisons.

References

1. R. D. Shannon, Revised effective ionic radii and systematic studies of interatomic distances in halides and chalcogenides, *Foundations of Crystallography*, 1976, **32**, 751–767.
2. N. E. Kirchner-Hall, W. Zhao, Y. Xiong, I. Timrov and I. Dabo, Extensive benchmarking of DFT+ U calculations for predicting band gaps, *Applied Sciences*, 2021, **11**, 2395.
3. T. Babuka, K. Glukhov, Y. Vysochanskii and M. Makowska-Janusik, New insight into strong correlated states realised in a ferroelectric and paraelectric chalcogenide Sn2P2S6 crystal, *RSC Advances*, 2017, **7**, 27770–27779.
4. P. Basera and S. Bhattacharya, Chalcogenide perovskites (ABS3; A= Ba, Ca, Sr; B= Hf, Sn): an emerging class of semiconductors for optoelectronics, *The Journal of Physical Chemistry Letters*, 2022, **13**, 6439–6446.
5. M. K. Horton, P. Huck, R. X. Yang, J. M. Munro, S. Dwaraknath, A. M. Ganose, R. S. Kingsbury, M. Wen, J. X. Shen and T. S. Mathis, Accelerated data-driven materials science with the Materials Project, *Nature Materials*, 2025, **24**, 1522–1532.
6. A. Jain, S. P. Ong, G. Hautier, W. Chen, W. D. Richards, S. Dacek, S. Cholia, D. Gunter, D. Skinner and G. Ceder, Commentary: The Materials Project: A materials genome approach to accelerating materials innovation, *APL materials*, 2013, **1**.
7. K. Huber, *Molecular spectra and molecular structure: IV. Constants of diatomic molecules*, Springer Science & Business Media, 2013.
